# Multi-Target Strategy for Pan/Foot-and-Mouth Disease Virus (FMDV) Detection: A Combination of Sequences Analysis, *in Silico* Predictions and Laboratory Diagnostic Evaluation

**DOI:** 10.3389/fvets.2018.00160

**Published:** 2018-07-12

**Authors:** Liliam Rios, Carmen L. Perera, Liani Coronado, Damarys Relova, Ana M. Álvarez, Llilianne Ganges, Heidy Díaz de Arce, José I. Núñez, Lester J. Pérez

**Affiliations:** ^1^Reiman Cancer Research Laboratory, Faculty of Medicine, University of New Brunswick, Saint John, NB, Canada; ^2^Centro Nacional de Sanidad Agropecuaria, OIE Collaborating Centre for Diagnosis and Risk Analysis of the Caribean Region, San José de las Lajas, Cuba; ^3^Instituto Nacional de Investigaciones Agricolas, Maracay, Venezuela; ^4^OIE Reference Laboratory for Classical Swine Fever, IRTA-CReSA, Barcelona, Spain; ^5^IRTA, Centre de Recerca en Sanitat Animal (CReSA, IRTA-UAB), Campus de la Universitat Autonoma de Barcelona, Barcelona, Spain; ^6^Hospital Italiano de Buenos Aires, Buenos Aires, Argentina; ^7^Dalhousie Medicine New Brunswick, Dalhousie University, Saint John, NB, Canada

**Keywords:** foot-and-mouth disease virus, multi-target strategy, sequences analysis, primer efficiency evaluation, entropy, amplicon stability

## Abstract

Foot-and-mouth disease (FMD) is a highly contagious viral disease affecting cloven-hoofed animals that causes severe economic losses. The disease is characterized by a vesicular condition and it cannot be differentiated from other vesicular diseases. Therefore, laboratory confirmation of any suspected FMD case is compulsory. Despite viral isolation in cell cultures has been considered for many years as the gold standard for FMD diagnosis, the advantages of real-time reverse transcription polymerase chain reaction (rRT-PCR) technology have motivated its use directly in clinical specimens for FMD diagnosis. The current work was aimed to develop and validate a molecular multi-check strategy using rRT-PCR (mMulti-rRT-PCR) based on *SYBR-Green I* for pan/foot-and-mouth disease virus (pan/FMDV) diagnosis. From *in silico* approaches, different primer pairs previously reported were selected and modified to reduce the likelihood of viral escape as well as potential failures in the pan/FMDV detection. The analytical parameters were evaluated using a high number of representative viral strains. The repeatability of the assay and its performance on field samples were also assessed. The mMulti-rRT-PCR was able to detect emergent FMDV strains that circulated in South America between the years 2006–2010 and on which the single rRT-PCRs failed when they were applied independently. The results obtained here showed that the proposed system is an accurate and rapid diagnosis method for sensitive and specific detection of FMDV. Thus, a validated mMulti-rRT-PCR assay based on *SYBR-Green I* detection coupled to melting curves resolution for pan/FMDV diagnosis on clinical samples is proposed. This study also highlights the need to incorporate the multi-target detection principle in the diagnosis of highly variable agents, specially, of those listed by OIE like FMDV.

## Introduction

Foot-and-mouth disease (FMD) is a highly contagious viral disease affecting cloven-hoofed animals including cattle, pigs, sheep, goats and around 70 wildlife species (1). Despite the high morbidity of the disease, mortality is low and mostly restricted to young animals (2). Nevertheless, FMD causes severe economic losses due to both significant decrease in production efficiency of the animals affected and sanitary control measures costs (3).

The causative agent of FMD is the FMD virus (FMDV), a single stranded positive sense RNA virus, belonging to the genus *Aphthovirus*, family *Picornaviridae*, order *Picornavirales*. The viral genome is about 8,400 nt in length and consists of a single open reading frame (ORF) flanked by two untranslated regions (UTRs). The ORF encodes four structural proteins (VP1, VP2, VP3, and VP4) and the non-structural proteins (Lab/b, 2A, 2B, 2C, 3A, 3B_1−3_, 3C, and 3D) (1). The virus replicates extremely rapid with a high mutation rate, generating viral populations with genetically diverse genomes known as viral *quasispecies* (4).

A consequence of the high variability of FMDV is the existence of seven different serotypes (O, A, C, Asia 1, SAT 1, SAT 2, and SAT 3) and further numerous variants that can be classified in topotypes (5). Serotypes Asia 1, SAT 1, 2, and 3 have a restricted geographical distribution, while serotypes A, O, and C, may occur in many different regions, even though, serotype C seems to have disappeared since it was last reported over 10 years ago (6).

The disease is characterized by a vesicular condition of the feet, oral cavity, and the mammary glands of females. However, these clinical signs cannot be differentiated from those caused by other viral vesicular diseases, like vesicular stomatitis and swine vesicular disease. Moreover, bovine viral diarrhea can produce mucosal lesions that can be confused with FMDV. Thus, laboratory confirmation of any suspected FMD case is compulsory. Laboratory diagnosis of FMD can be performed either by detecting the virus or any of its components or by demonstrating the presence of virus-specific antibodies in samples of tissue or fluid (7). Despite viral isolation in cell cultures has been considered for many years as the gold standard for FMD diagnosis, the advantages of real-time reverse transcription polymerase chain reaction (rRT-PCR) technology have motivated its use on clinical specimens (8). The World Organization for Animal Health (OIE) has approved the use of rRT-PCR assays as conventional diagnostic methods (9, 10). However, these assays are based on Taqman technology, which rely heavily on primers and probes flanking a single region of the FMDV genome, making them susceptible to false negative results when new variants emerge (11, 12).

To overcome this drawback, different strategies for viral RNA detection have been developed, including: multiplex rRT-PCR arrays based on *SYBR-Green I* [(12), minor groove binder (MGB) probe real-time RT-PCR (13)], linear-after-the-exponential polymerase chain reaction (LATE-PCR) (14, 15) and novel isothermal amplification approaches such as reverse transcription loop-mediated isothermal amplification (RT-LAMP) (16–18) insulated-isothermal (RT-iiPCR) (19) and reverse transcription recombinase polymerase amplification (RT-RPA) (20). Nevertheless, the fluorescence-based multiplex assay already described uses a total of 36 primer pairs, making it highly laborious and increasing the cost of the method in the practice (12). The LATE-PCR assays are very efficient symmetric PCR methods (21, 22) but with the disadvantage that the detection of this assay is based on a high-melting temperature (Tm) molecular beacon probes, which are prone to fail with single nucleotide variations [reviewed in Belák et al. (11)]. Therefore, its use in cases of FMD outbreaks caused by novel emergent viral strains could be limited. For RT-LAMP assays, a perfect hybridization of six primers is needed to yield a successful amplification reaction (23), which is very difficult to obtain in RNA viruses, especially in those highly variable (24). In addition, only the rRT-PCR assays have demonstrated enough sensitivity to detect persistently infected animals (carriers) with FMDV (25).

Therefore, the current work was aimed to develop and validate a molecular multi-check strategy using rRT-PCR (mMulti-rRT-PCR) based on *SYBR-Green I* for pan/foot-and-mouth disease virus (pan/FMDV) diagnosis.

## Materials and methods

### Viruses and samples

#### Viruses

All strains, field isolates and genetic material used in this study, which included a representation of all seven serotypes of FMDV, viral isolates of vesicular stomatitis virus (VSV) serotypes Indiana and New Jersey, genetic material of swine vesicular disease virus (SVDV), representative strains for all three genotypes of classical swine fever virus (CSFV), representative strains for two species of bovine viral diarrhea virus (BVDV), border disease virus genotype 1 (BDV) and encephalomyocarditis virus (EMCV), are listed in Table 1.

**Table 1:** Summary of viruses and genetic material used in this study^a^.

**Virus**	**serotype/genotype**	**Number of strains**	**Multi-rRT-PCR assay**
FMDV	A	36	Positive
	O	12	Positive
	C	1	Positive
	SAT 1	1	Positive
	SAT 2	1	Positive
	SAT 3	1	Positive
	Asia 1	1	Positive
VSV	New Jersey	89	Negative
	Indiana	8	Negative
CSFV	Genotype 1	1	Negative
	Genotype 2	1	Negative
	Genotype 3	1	Negative
BVDV	Genotype 1	1	Negative
	Genotype 2	1	Negative
BDV	Genotype 1	2	Negative
EMCV		1	Negative
SVDV		1	Negative

a *A complete and detailed list containing all viruses and genetic material used is shown in Table S1*.

#### Cell culture and virus propagation

Viruses were grown and titrated in PK15 cell line (ATCC® CCL-33™) (CSFV strains), MDBK cell line (ATCC® CCL-22™) (BVDV and BDV strains) and BHK-21 (ATCC® CCL-10™) (FMDV and VSV strains/isolates) following standard procedures described in the OIE Manual (26–30). The Cuban EMCV 744/03 strain (31) was propagated on BHK-21 cells in MEM supplemented with 5% FBS as described in Diaz de Arce et al. (32), briefly:after approximately 16 h of incubation at 37°C, when more than 80% of the cells showed cytopathology, the cells were subjected to three freeze–thaw cycles. Non-infected PK15, MDBK, and BHK-21 cells were also included in the specificity assays. The cell cultures and media were previously tested to be free of contaminating pestivirus.

#### Ethics statement

A collection of 40 tissue homogenate samples (epithelium and vesicular fluids) from cattle that presented a variety of clinical signs including skin lesions and vesicles, collected in the outbreaks during 2006–2010 described, was used to assess the performance of the mMulti-rRT-PCR based on SYBR-Green I system. International standards for animal welfare were addressed for all animal samples collected, following the regulations for animal sampling of the Bioethics and Biosafety Code of the Republic of Venezuela, MPPCYT-FONACIT.

#### Virus isolation

The virus isolation (VI) method used to identify FMDV was performed on BHK-21 cells following the standard procedures of the OIE Manual (27). The cell cultures were examined for cytopathic effect (CPE).

### Primers selection

Four primer pairs reported in the scientific literature for the specific detection of pan/FMDV were selected (Table 2). The selection included the two primer pairs from the assays recommended for FMDV detection based on rRT-PCR by OIE Manual (9, 10), the primer pair from the rRT-PCR assay reported by Moniwa et al. (33) and the primer pair from the conventional RT-PCR assay reported by Sáiz et al. (34). This last primer pair was included because the performance of the assay was comparable to the results obtained from other rRT-PCR assays in an inter-laboratory comparison trial to evaluate virus isolation and RT-PCR detection methods for FMDV (35). The primers selected were verified for their thermodynamic properties, secondary structures and primer-primer interactions using OligoExplorer v1.2 and OligoAnalyzer v1.1.2 softwares (Gene LinkTM, USA). The specificity of the sequences was determined by comparison to the GenBank database using BLASTn searches configured for short, nearly exact matches (word size 7, expect value 1,000).

**Table 2:** Assays for pan/FMDV detection out of which the primer pairs were selected for the development of mMulti-rRT-PCR assay.

**Primer/probe**	**Sequence (5^′^-3^′^)**	**Genome position (5^′^-3^′^)^c^**	**Identification**	**Original source**
SA-IR-219-246F	CACYTYAAGRTGACAYTGRTACTGGTAC	896-923	FP-TgR1	Reid et al. (9)
SA-IR-315-293R^a^	CAGATYCCRAGTGWCICITGTTA	970-992	RP- TgR1	Reid et al. (9)
SAmulti2-P-IR-292- 269R^b^	CCTCGGGGTACCTGAAGGGCATCC	946-969		Reid et al. (9)
6769^mod^	ACTGGGTTTTACAAACCTGTGA	7866-7887	FP- TgR2	Callahan et al. (10)
6875^mod, a^	GCGAGTCCTGCCACGGA	7956-7972	RP- TgR2	Callahan et al. (10)
6820^b^	TCCTTTGCACGCCGTGGGAC	7917-7936		Callahan et al. (10)
**FMDV**-3D forward	ACTGGGTTTTAYAAACCTGTGATG	7866-7889	FP- TgR3	Moniwa et al. (33)
**FMDV**-3D reverse^a^	TCAACTTCTCCTGKATGGTCCCA	7931-7953	RP- TgR3	Moniwa et al. (33)
**FMDV**-3D TaqMan-probe^b^	ATCCTCTCCTTTGCACGC	7911-7928		Moniwa et al. (33)
A^a^	CACACGGCGTTCACCCA(A/T)CGC	8060-8080	RP- TgR4	Sáiz et al. (34)
B^mod^	GACAAAGGTTTTGTTCTTGGTC	7791-7812	FP- TgR4	Sáiz et al. (34)

a *Reverse primer*.

b *Probe*.

c *Genome position according to FMDV OV1/UK/24 (Accession no. AY593829).^mod^ Since the results obtained from Bioinformatics analysis the primers were modified in this study. The modified primers were used for all the applications of the current study including: conventional RT-PCR, single rRT-PCRs and mMulti-rRT-PCR*.

### Bioinformatic analysis of FMDV target regions (TgR)

#### Sequence datasets and multiple alignment

All 328 unique sequences of FMDV complete genome available at GenBank Database were downloaded and used in the analysis (Table S2). A sequence analysis based on multiple alignments for all sequences downloaded was used to examine how well the primers selected (probe sequences from the rRT-PCR selected (Table 2) were also included) matched at the target sequences. Multiple alignment among FMDV sequences and primer pairs was performed using the algorithm Clustal W method included in the BioEdit version 7.2.5 program (36).

#### Primer efficiency and theoretical melting temperature of the amplicon sequences

The primer analysis software Oligo 7.6 (Molecular Biology Insights, USA) was used to determine the priming efficiency of each primer selected to the target site on the FMDV sequence (Table S2). This computational tool ponders mismatches, duplex stability, bulge loops, and the distance of these elements from the primer's 3′end. This software considers, a priming efficiency number (P.E. #) of 170 as false priming and P.E.#>210 may result in priming during PCR or cycle sequencing conditions, therefore the cut off was set to P.E.#>210 as previously described by Pérez et al. (37).

The theoretical melting temperatures (Tm) of the amplicon sequences were also estimated using the Oligo v7.6 software package (Molecular Biology Insights, USA).

#### Variability of the target regions and amplicons stability analyses

The variability of the TgRs, in special the matching regions of the primers and probes on the FMDV sequences, was assessed by plotting the nucleotide sequence entropy calculated by the DAMBE program (38). The value 0.1 was established as the stability cutoff value. The stability of each amplicon was estimated by Gibbs free energy (ΔG) for each FMDV sequence, calculated by the Oligo v7.6 software package (Molecular Biology Insights, USA). The media values of ΔG and nucleotide sequence entropy for each amplicon were plotted using GraphPad Prism v6.0 (GraphPad software, Inc.).

### RNA isolation and cDNA synthesis

Total RNA was isolated from supernatant cell culture, epithelium and vesicular fluids using QIAamp Viral RNA Mini Kit (Qiagen GmbH) following the manufacturer's directions, briefly:in all cases, an initial volume of 150 μL of the sample was used to obtain a final volume of 50 μL of RNA diluted in nuclease-free water and stored at−80°C. The cDNA was synthetized using random primers and M-MLV reverse transcriptase, as described by Díaz de Arce et al. (32).

### Simple rRT-PCRs and mMulti-RT-PCR

All rRT-PCR experiments were conducted on a LightCycler 2.0® instrument (Roche Applied Science, Mannheim, Germany). During the optimization of the protocols several experimental steps were conducted to set up the reagent concentrations and the thermocycling parameters (data not shown). The assays were first optimized in single format and finally optimized as a mMulti-RT-PCR. Thus, the amplification reactions were conducted in a 20μL glass capillary containing 5μL of cDNA, 0.5μL of each of the forward and reverse primers (final concentration: 0.3μM (FP/RP-TgR2 and FP/RP-TgR3), 0.4μM FP/RP-TgR1 and/or 0.5μM FP/RP-TgR4), 2μl of LightCycler^®;^ FastStart DNA master *SYBR-Green I* (10X) (Roche Diagnostics, Manheim, Germany), 2μL of MgCl_2_ (final concentration 3.5 mM) and enough nuclease-free water (Promega, Madison, WI, USA) for a final volume of 20μL for each reaction.

A touchdown thermal profile for both single and mMulti-rRT-PCR assays was used for all primer sets. This strategy facilitates the amplification of the specific targets avoiding the phenomenon of preferential amplification. Thus a general thermal profile was selected as follows: 95°C for 10 min followed by 40 cycles of 95°C for 10 s, a touchdown annealing from 60°C to 55°C for 5 s, with a step size of 0.5°C and a step delay of 1 cycle, followed by 72°C for 20 s with acquisition of fluorescent data. After the PCR cycles, a melting curve analysis was included (0 s at 95°C, 15 s at 65°C, 0 s at 95°C) to distinguish between the specific and non-specific amplification products. The melting peaks were visualized by plotting the first negative derivative of the fluorescence over temperature vs. the temperature (–dF/dT vs. T) produced by the LightCycler® Software 4.05.415 (Roche Applied Science, Mannheim, Germany). The Tm value was defined as the peak of the curve obtained. This methodology has been previously applied and described by our research group for the development of rPCR diagnostic tools for other viral agents (39, 40).

### Conventional RT-PCR

Single specific end-point conventional RT-PCR assays were conducted in a 50 μL reaction volume that included 2 μL of the cDNA sample, 1xGoTaq® Flexi DNA Polymerase (Promega, Madison, WI, USA) (200μM of each dNTP, 1.5 mM MgCl_2_, pH 8.5) and 1 μM of each primer (Table 2). The PCR reaction was performed in a thermal cycler (Eppendorf, Mastercycler) using the following thermal-profile: one cycle of 2 min at 95°C followed by 35 cycles of denaturation for 30 s at 95°C, annealing for 30 s at 55°C and elongation for 30 s at 72°C. After the cycling step a final extension of 5 min at 72°C was included. In addition, to compare the performances of mMulti-rRT-PCR and the single rRT-PCR assays on field samples, the conventional RT-PCR described by Rodríguez et al. (41) was accomplished.

### *In vitro* transcription reaction

To determine the limit of detection of the assays in terms of RNA copy numbers, *in vitro*-transcribed RNA of the four targeted regions were obtained. The total RNA of FMDV/Isolate 21928/serotype O/VEN/2009 isolate was obtained, and the four targeted regions were amplified by RT-PCR using the four primer pairs selected (Table 2). the methodology used was previously described in Pérez et al. (37), briefly:in every cases the reverse primers were coupled to a T7 promoter. The amplification products were directly *in vitro*-transcribed from the polymerase promoter site T7 using a MEGAscript-Kit (Ambion) following the manufacturer's directions. Spectrophotometry was used to quantify the RNA transcripts obtained, and the precise number of RNA molecules was determine suing the formula described by Fronhoffs et al. (42). Thus, a total of 1.6 × 10^13^ RNA copies/μL was obtained in each case.

### Analytical sensitivity

The analytical sensitivities or limits of detection (LoD) of the single and mMulti r-RT-PCR assays were estimated by evaluating serial 10-fold dilutions in nuclease-free water of quantified standard RNA (obtained as described in 2.7). The dilutions assessed were ranged from 10^9^ RNA copies/μL to 10^−1^ RNA copies/μL for each TgR. The LoD of the assays was determined from three independent replicates. In the estimation of the analytical sensitivity of the assays the same experimental conditions described by Pérez et al. (37) were considered. Standard curves based on different RNA copies were generated, and the reaction efficiencies were obtained using LightCycler software (Version 4.05).

### Analytical specificity

The specificity of the mMulti-rRT-PCR was assessed by the analysis of viral RNA extracted from FMDV and other viruses. This last group included viruses causing vesicular disease resembling FMDV (VSV and SVDV), a virus affecting pigs that does not cause vesicular disease but is genetically related to FMDV (EMCV) and other RNA viruses that could be concomitant in the same clinical sample depending of the animal host sampled (CSFV for pigs, BVDV for cattle and BDV for sheep and goats), all of them included in Table 1. Each viral strain or isolate used was tested in triplicate.

### Influence of ramp time rates on the melting curves

The impact of the melt rate on the resolution of the Tm-value of the simultaneously amplified amplicons was assessed for the mMulti-rRT-PCR assays by testing the different melt rates associated with the acquisition of fluorescent data as previously described (36). Briefly: three different melt rates were tested: 0.1, 0.4, and 0.8°C/s.

### Intra- and inter-assay variability

The repeatability of the mMulti-rRT-PCR assay was calculated using three different concentrations:strong positive (10^6^ RNA copies/μL), medium positive(10^4^ RNA copies/μL) and weak positive(10^2^ RNA copies/μ). The intra-assay variability was assessed by evaluating each dilutionin triplicate. Whereas the inter-assay variability, was determine by analyzing each condition in three independent runs conducted by two operators on different days. The standard deviation (SD) was calculated by LightCycler® Software 4.05.415 (Roche Applied Science, Mannheim, Germany), and the coefficient of variation (CV) was obtained using the formula CV = SD [Ct–value]/overall mean[Ct–value]) × 100, following previously reported guidelines (U.S. Environmental Protection Agency, 2004).

### Interpretation of results

The samples that yielded a Ct value below 35 with a curve in the amplification plot that displayed an exponential increase and a specific dissociation temperature for any of the four TgR (TgR1, Tm = 82.5°C ± 0.3°C; TgR2 Tm = 85.4°C ± 0.5°C; TgR3, Tm = 84.9°C ± 0.1°C, and TgR4, Tm = 88.0°C ± 0.3°C) were considered positive for FMDV. Thus, mMulti-rRT-PCR assay was considered positive when at least one of the target regions yielded a positive result.

### Statistical analysis

The significant differences among the means of the Tm-values corresponding to the different matrices tested (nuclease free water, epithelium and vesicular fluid) were analyzed by one-way analysis of variance (ANOVA) using GraphPad Prism v6.0 (GraphPad software, Inc.). In all cases a *p*-value < 0.05 was considered statistically significant. In addition, the media values and standard errors of ΔG and global entropy for each amplicon were statistically analyzed using GraphPad Prism v6.0 (GraphPad software, Inc.,). In all cases a *p*-value < 0.05 was considered statistically significant.

## Results

### Bioinformatic analysis of primers, probes and TgRs

**Figure 1:**
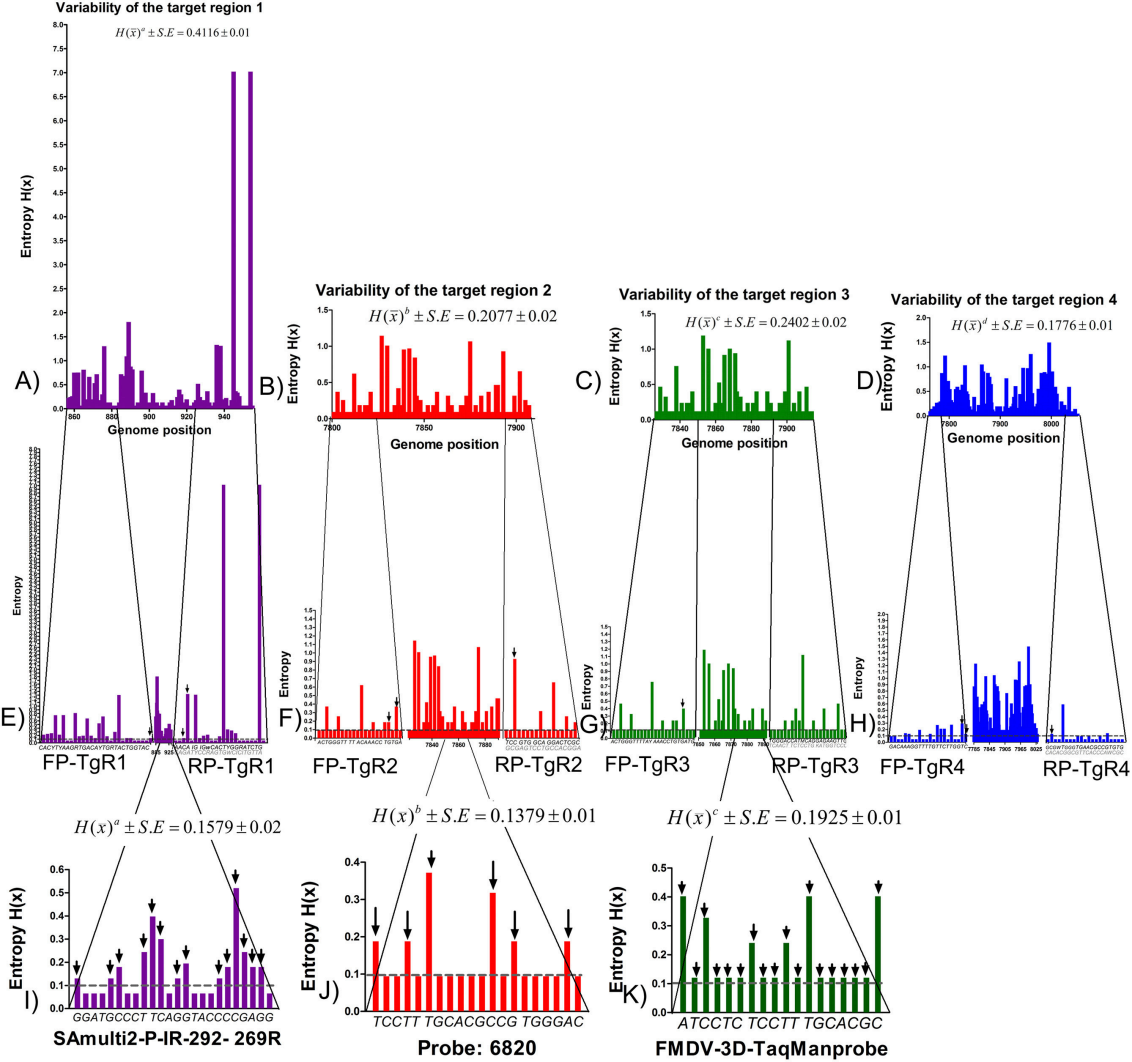
Analysis of variability of the TgRs selected for the detection of FMDV. Entropy plots obtained from 328 sequences of FMDV for each TgR are shown, **(A)** TgR1, **(B)** TgR2, **(C)** TgR3, and **(D)** TgR4. The global entropy value estimated by the entropy media value and standard error associated to each TgR is also shown. Different letters mean statistical differences (*p* < 0.05). Entropy plots for each primer of each TgR are also shown **(E)** FP/RP-TgR1, **(F)** FP/RP-TgR2, **(G)** FP/RP-TgR3, and **(H)** FP/RP-TgR4; the sequences for each forward and reverse primer are denoted, critical positions for the last four nucleotides at the 3′OH of each primer are indicated with black arrows. Entropy plots for the probe region obtained from the assays reported by Reid et al. (9), Callahan et al. (10), and Moniwa et al. (33) are shown, **(I)** probe matching TgR1, **(J)** probe matching TgR2, and **(K)** probe matching TgR3. The global entropy value estimated by the entropy media value and standard error associated to each TgR is also shown. The sequences of each probe are denoted and critical positions (*H* > 0.1) for each probe are indicated with black arrows.

The analysis of variability of the complete genome of FMDV revealed several areas highly variable within the viral genome, represented by high values of entropy and other more conserved regions, represented by low values (data not shown). For the TgRs selected in the current study, the more conserved regions were the TgRs (TgR2-TgR4) included within the viral polymerase region. Their mean global entropy values ranged between 0.1776 and 0.2402, being the TgR4 the most conserved, with a mean global entropy value of 1.1776. The TgR1, which is included into the *IRES* region was the most variable, with a mean entropy value of 0.4116 (Figure 1). Analyzing the primer binding sites, several variable positions were found, which could produce mismatches within the target sequences (Figure 1). Thus, 26 variable sites represented by high entropy values were found for the primer pair FP/RP-TgR1, 13 for the primer pair FP/RP-TgR2, 15 for the primer pair FP/RP-TgR3, and 12 for the primer pair FP/RP-target 4. Nevertheless, taking into account that for an effective priming event between the target sequence and the primers, the critical nucleotides are those located at the 3 ′OH extreme, these positions were analyzed in more detail. Inspecting the entropy values for the last four nucleotides at the 3 ′OH of the primers, it was observed that, excepting the primer pair FP/RP-TgR3, the rest showed three nucleotides with values greater than 0.1, proving they are highly susceptible to priming failure (Figure 1).

Concerning to the probe binding regions, the lowest global entropy mean value was obtained for the probe included into TgR2 proposed by Callahan et al. (10) (0.1379) (Figure 1), whereas the highest mean global entropy value was obtained for the probe in TgR3 proposed by Moniwa et al. (33) (0.1925) (Figure 1). Inspecting the critical positions represented by high entropy values, 14 for the probe included in TgR1 and 6 for the probe in TgR2 were found, whereas all sites for the probe included in TgR3 were surprisingly variable with entropy values higher than 0.1, suggesting a high instability in this region. Taking into account that a single mismatch in short TaqMan probes (18–20 nucleotide length) has an effect on both the Tm and the sensitivity (decreasing around 9°C and 0.7 Log, respectively) (43), together with the high stringency of the assays selected (10, 44), then one mismatch between probe and target (probe TgR2 and TgR3) was considered as cut off for failures in the detection of the FMDV sequences analyzed. In the case of the probe targeting region 1, the cut off was established in two mismatches because the length of this probe is 24 nt (near to 25 nt) and it would be affected by two or more mismatches (40). After these considerations, several potentially undetected sequences by the probes of the assays selected were found: 58 sequences for the probe targeting region 1, 13 sequences for the probe targeting region 2 and 31 for the probe targeting region 3 (Figure S1). All these potential detection failures caused by hybridization between the probe and the target sequences were avoided in our assay since a *SYBR-Green I* format was used.

The *in-silico* evaluation of the primer efficiency showed a low value (170 < P.E. < 210) or false priming (P.E. < 170) for 11 FMDV sequences by the primer pair FP/RP-TgR2 and for 9 FMDV sequences by the primer pair FP/RP-TgR4 (Table 3 and Table S3). The two remaining primer pairs used in this study yielded high P.E. values for all 328 sequences analyzed (Table 3 and Table S3).

**Table 3:** Summary of the results of priming efficiency (P.E.) values for each primer pair on the FMDV sequences (P.E. for the primer against a linked sequence/maximal theoretical P.E for a perfect match); low P.E. value (170 < P.E. < 210) or false priming (P.E. < 170) were denoted in bold and cursive letters (the original table showing the results obtained from the evaluation of the all sequences can be find in Table S2).

**GenBank access number| Strain or isolate**	**Primer pair**			

	**FP/RP-TgR1**	**FP/RP-TgR2**	**FP/RP-TgR3**	**FP/RP-TgR4**
AY593829.1| FMDV O isolate o6pirbright iso58	(464/487)/(516/516)	(458/458)/(469/469)	(474/474)/(482/482)	***(153/454)/(587/587)***
AY593788.1| FMDV A isolate abrazil iso67	(385/479)/(440/440)	***(458/458)/(181/469)***	(474/474)/(482/482)	(454/454)/(587/587)
AY593787.1| FMDV A isolate abage iso63	(385/479)/(457/457)	***(458/458)/(181/469)***	(474/474)/(482/482)	(454/454)/(587/587)
AY593776.1| FMDV A isolate a3mecklenburg iso81	(496/496)/(516/516)	(458/458)/(392/469)	(474/474)/(482/482)	***(146/454)/(587/587)***
AY593766.1| FMDV A isolate a23kenya iso8	(447/487)/(466/466)	(458/458)/(415/469)	(474/474)/(482/482)	***(176/454)/(587/587)***
AY593759.1| FMDV A isolate a1bayern iso41	(487/487)/(516/516)	(458/458)/(331/469)	(474/474)/(482/482)	***(153/454)/(587/587)***
AY593843.1| FMDV SAT 1 isolate sat16swa iso16	(363/492)/(457/457)	***(458/458)/(200/469)***	(474/474)/(410/494)	(454/454)/(587/587)
AY593839.1| FMDV SAT 1 isolate sat120 iso11	(363/492)/(416/416)	***(178/458)/(469/469)***	(326/474)/(494/494)	(454/454)/(371/587)
DQ404164.1| FMDV O strain UKG/11676/2001	(487/487)/(516/516)	(458/458)/(469/469)	(474/474)/(482/482)	***(153/454)/(587/587)***
HQ832576.1| FMDV A isolate IND 21/1990	(487/487)/(445/516)	(458/458)/(469/469)	(474/474)/(482/482)	***(176/454)/(587/587)***
KC412634.1| FMDV Asia 1 isolate Asia1/HN/2006	(487/487)/(516/516)	***(178/458)/(469/469)***	(326/474)/(482/482)	(454/454)/(587/587)
JX040486.1| FMDV O isolate BUL/11/2011	(362/487)/(390/444)	(458/458)/(469/469)	(474/474)/(482/482)	***(175/454)/(587/587)***
EF614458.1| FMDV Asia 1 strain Asia1/MOG/05	(487/487)/(516/516)	***(178/458)/(469/469)***	(326/474)/(482/482)	(454/454)/(587/587)
HQ631363.1| FMDV Asia 1 isolate Asia1/1/YZ/CHA/06	(487/487)/(516/516)	***(178/458)/(469/469)***	(326/474)/(482/482)	(454/454)/(587/587)
FJ906802.1| FMDV Asia 1 strain Asia1/WHN/CHA/06	(499/499)/(516/516)	***(178/458)/(469/469)***	(326/474)/(482/482)	(454/454)/(587/587)
GU931682.1| FMDV Asia 1 isolate Asia1/YS/CHA/05	(487/487)/(516/516)	***(178/458)/(469/469)***	(326/474)/(482/482)	(454/454)/(587/587)
DQ989323.1| FMDV Asia 1 isolate IND 9703	(487/487)/(466/466)	(458/458)/(469/469)	(474/474)/(482/482)	***(153/454)/(587/587)***
EF149009.1| FMDV Asia 1 strain Asia 1/Jiangsu/China/2005	(499/499)/(516/516)	***(141/458)/(469/469)***	(279/474)/(482/482)	(454/454)/(587/587)
KC462884.1| FMDV Asia 1 isolate Asial/HN/2006	(487/487)/(516/516)	***(178/458)/(469/469)***	(326/474)/(482/482)	(454/454)/(587/587)

To assure the primer pairs FP/RP-TgR2 and FP/RP-TgR4 would perfectly match on the sequences they failed to detect as well as on other FMDV viral strains that could exist in nature with the same sequence background, these primer pairs were modified. Modifications are shown in bold and included the introduction of degenerated nucleotides: **FP-TgR2:** 5′-ACTGGGTTTTACAAACCTGT**R**A-3′/**RP-TgR2**: 5′-GCGAGTCCTGCCAC**V**GA-3′; FP**-TgR4:** 5′- GACAAAGGTT TTGTTCTTGG**IS**-3′/**RP-TgR4:** 5′-CACACGGCGTTCACCCAWCGC-3′.

### mMulti-rRT-PCR pan/FMDV

**Figure 2:**
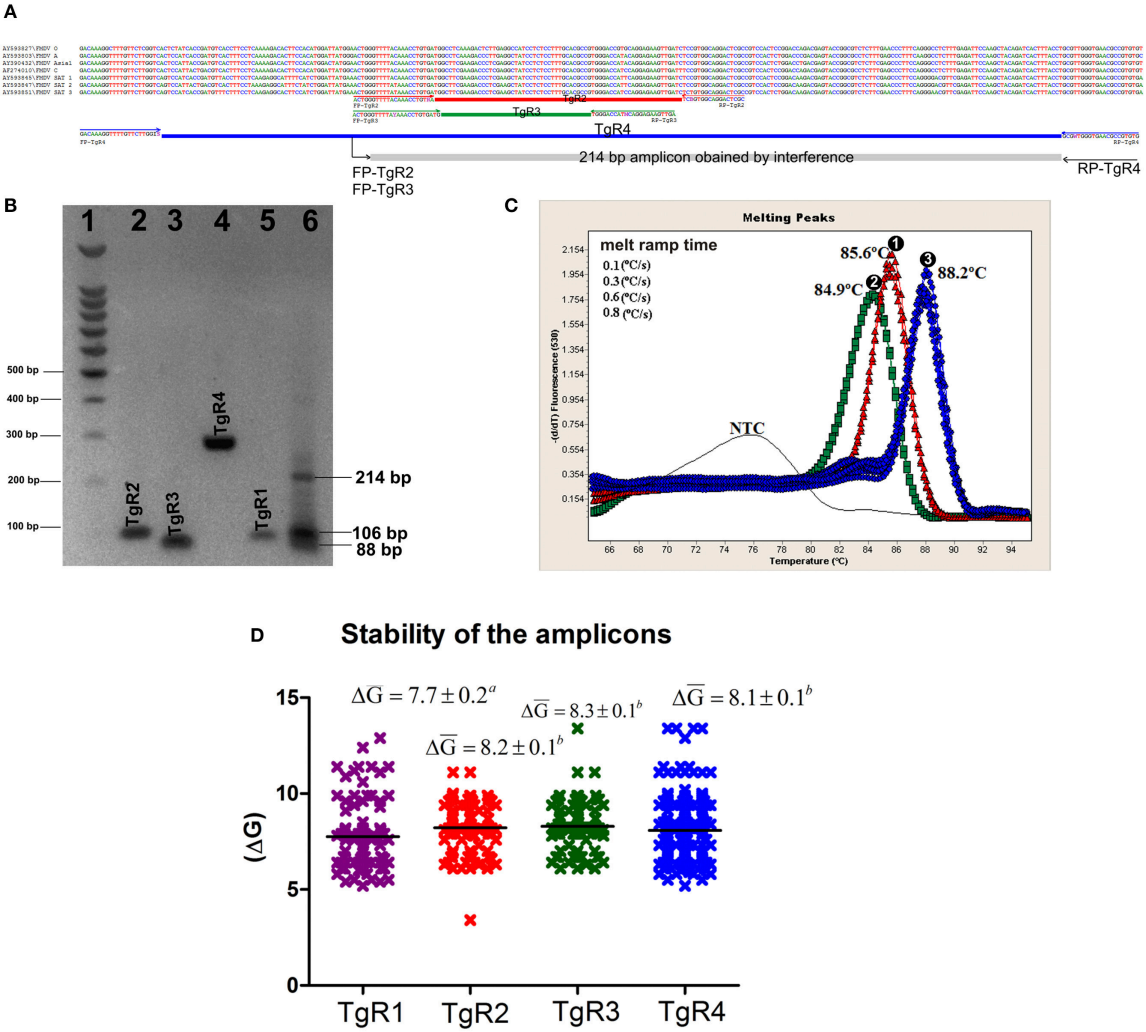
*In silico* and *in vitro* evaluation of the amplification products. **(A)** Alignment of representative sequences of the seven serotypes of FMDV and primer pairs of TgR2, TgR3, and TgR4. The sequence and orientation of each primer is shown. Each amplicon is represented by a colored rectangle, red for TgR2, green for TgR3, blue for TgR4 and gray for the amplicon obtained from the interference of the forwards primes FP-TgR2 and FP-TgR3 with RP-TgR4, the size of this amplicon is also denoted. **(B)** Electrophoresis in 2% agarose gel, each amplification product obtained is shown, 1- molecular weight marker 100 bp (Promega, Madison, WI, USA); 2- amplicon of the TgR2; 3- amplicon of the TgR3; 4- amplicon of the TgR4, 5- amplicon of the TgR1; 6- amplification products obtained from mix of primer pairs of TgR2, TgR3, and TgR4, the size of the amplification product obtained from of the forwards primes FP-TgR2 and FP-TgR3 with RP-TgR4 is shown, the size of the amplicon is also denoted. For sizes of the amplicons of TgR 1-4 see Table 3 **(C)** Melting curve analysis of the amplification products obtained from the combination of: 1- primer pair TgR1+ primer pair TgR2, 2- primer pair TgR1+ primer pair TgR3 and 3- primer pair TgR1+ primer pair TgR4 are shown; the specific Tm obtained is also denoted. **(D)** Stability of the amplification product for each TgR, defined by ΔG media value and standard error associated to each TgR are shown, different letters mean statistical differences (*p* < 0.05).

According to the BLASTn results, all primer sequences were specific for their respective targets (data not shown). From the *in-silico* analysis for each amplicon, theoretical media values of Tm were estimated, this analysis clearly showed difference in the Tm of each amplification product, excepting products from TgR1 and TgR3, which showed a difference less than 1°C (Table 4). Nevertheless, from the *in vitro* transcripts evaluation and the posterior feasibility analysis on epithelium matrix assessment, using the single rRT-PCR assays, the practical Tm-values for each amplicon showed that all amplification products were clearly discriminated by melting curve analysis, excepting TgR2 and TgR3 which had a difference in Tm-values of less than 1°C (Table 4). Therefore, to simplify the assay and decrease the costs, different combinations among the four primer sets were assessed. The results obtained showed changes on the specific signal of amplification caused by the translocation effect of SYBR-Green I among the different amplicons. Analyzing the parameters that could cause this effect, it was observed that the amplification of products of interference and the difference in the stability of the specific amplicons difficulted the differentiation of the products when co-amplifications were conducted in the same vessel of reaction (Figure 2). Avoiding these difficulties and considering the principle of Multi-PCR described by Belák et al. (11) the system was not forced to co-amplify the different TgRs in a single reaction, but it was rather developed to amplify the various TgRs side-by-side on the same run (mMulti-rRT-PCR).

**Table 4:** Evaluation of Tm-values from theoretical and practical analyses using the primer pairs selected for the detection of pan/FMDV.

**Identification**	**Genome region**	**Total sequences**	**Amplicon (bp)**	**%GC (~total GC)**	**Tm** ± **S.D (**^**°**^**C)**		

					**Theoretical**	**N.F.W**	**Epithelium**
FP/RP-TgR1	5′NTR	328	97	52.2 (50)	83.6 ± 0.6	82.5 ± 0.3	82.5 ± 0.3
FP/RP-TgR2	3DPol	328	106	54.6 (58)	85.1 ± 0.6	85.4 ± 0.5	85.4 ± 0.5
FP/RP-TgR3	3DPol	328	88	51.8 (46)	82.9 ± 0.6	84.9 ± 0.1	84.9 ± 0.1
FP/RP-TgR4	3DPol	328	290	52.3 (152)	87.1 ± 0.4	88.0 ± 0.3	88.0 ± 0.3

### Sensitivity, dynamic range, and linearity

The performance of the mMulti-rRT-PCR *SYBR-Green I* assay was determined using 11 different 10–fold serially diluted RNA transcripts. Each dilution was subjected to the assay in triplicate. For all TgRs the reactions generated positive amplification signals with specific melting curves until 10 RNA copies/μL (Figures S2A–D). Moreover, the reaction conducted on TgR4 also detected the dilution containing 1 RNA copy/μL (Figure S2D).

For each TgR, standard curves were generated by plotting the threshold cycle (Ct) values against the different copy numbers of RNA transcripts (Figure S3). Amplification efficiencies (E) ranging between 111 and 95.2% (Figure S3) were obtained for the different TgRs. Thus, the lowest E value was yielded by the TgR1 (Figure S3A), whereas the highest E value was obtained by TgR2 (Figure S3B). The linear dynamic range for the reactions accomplished on TgR1-TgR3 spanned within 10^8^-10^1^ gene copies/μL (Figures S3A–C), whereas the linear dynamic range for the reaction accomplished on TgR4 spanned within 10^6^-10^0^ RNA copies/μL (Figure S3D).

### Analytical specificity

The specificity of the mMulti-rRT-PCR system was tested on 158 genomic RNAs from a panel that included viral strains, viral isolates and genetic material (Table 1). The mMulti-rRT-PCR system was able to detect all 52 RNAs from FMDV strains, isolates and genetic material (Table 1), showing the four specific dissociation curves for each one of the TgRs detected (Tm_TgR1_ = 82.5 ± 0.3°C; Tm_TgR2_ = 85.4 ± 0.5°C; Tm_TgR3_ = 84.9 ± 0.1°C and Tm_TgR4_ = 88.0 ± 0.3°C). Amplification curves were not obtained with any of the other 152 RNA samples from non-FMDV strains, isolates or genetic material tested (Table 1).

### Intra- and inter-assay variability

The mMulti-rRT-PCR demonstrated a high repeatability with coefficients of variation (CV) within runs for each TgR of less than 2.0% (intra-assay variability) (Table S3). Thus, the higher CV percentage value was yielded by TgR4 at low RNA loads (Table S3). Taking this percentage value as the maximum intra-variability for the mMulti-rRT-PCR assay, then the assay proposed showed an intra-assay variability of 0.34% and between runs (inter-assay variability), the mMulti-rRT-PCR ranged from 0.92 to 1.90%.

### Application of mMulti-rRT-PCR assay to field samples

The results yielded by the mMulti-rRT-PCR, VI, every rRT-PCR assay considered separately and the conventional RT-PCR assay of the 40 field samples tested are shown in Table 5. A total of 26 samples (all from clinically suspected FMDV-infected animals) were positive for FMDV (65.0%) as detected by the mMulti-rRT-PCR system (Table 5).

**Table 5:** Performance of the mMulti-rRT-PCR system, single rRT-PCR assays, VI and conventional RT-PCR on field samples.

**Code**	**Sample**	**TgR1 (Ct)**	**TgR2 (Ct)**	**TgR3 (Ct)**	**TgR4 (Ct)**	**mMulti-RT-PCR**	**VI**	**RT-PCR^a^**
S001	Epithelium	Neg	Neg	Neg	Neg	Neg	Neg	Neg
S002	Epithelium	Neg	Neg	Neg	Neg	Neg	Neg	Neg
S003	Epithelium	Neg	24.73	Neg	20.25	Pos	Pos	Neg
S004	Epithelium	Neg	Neg	Neg	Neg	Neg	Neg	Neg
S005	Epithelium	33.82	Neg	Neg	26.75	Pos	Neg	Neg
S006	Epithelium	24.01	Neg	Neg	18.25	Pos	Pos	Pos
S007	Epithelium	Neg	Neg	Neg	Neg	Neg	Neg	Neg
S008	Vesicular fluids	21.25	25.68	29.11	22.75	Pos	Pos	Pos
S009	Epithelium	32.15	Neg	Neg	28.25	Pos	Neg	Neg
S010	Vesicular fluids	29.7	18.09	24.59	21.79	Pos	Pos	Pos
S011	Epithelium	Neg	Neg	Neg	Neg	Neg	Neg	Neg
S012	Vesicular fluids	25.08	19.83	Neg	23.11	Pos	Pos	Pos
S013	Epithelium	16.68	10.86	11.22	11.65	Pos	Pos	Pos
S014	Epithelium	Neg	21.23	21.68	18.25	Pos	Pos	Neg
S015	Epithelium	20.80	17.59	18.45	18.25	Pos	Pos	Pos
S016	Epithelium	15.63	10.86	11.35	11.75	Pos	Pos	Neg
S017	Epithelium	18.45	13.43	15.21	14.22	Pos	Pos	Pos
S018	Epithelium	Neg	Neg	Neg	Neg	Neg	Neg	Neg
S019	Epithelium	Neg	Neg	Neg	Neg	Neg	Neg	Neg
S020	Epithelium	Neg	Neg	Neg	Neg	Neg	Neg	Neg
S021	Epithelium	Neg	Neg	Neg	Neg	Neg	Neg	Neg
S022	Epithelium	Neg	Neg	Neg	Neg	Neg	Neg	Neg
S023	Epithelium	Neg	Neg	Neg	Neg	Neg	Neg	Neg
S024	Epithelium	Neg	Neg	Neg	Neg	Neg	Neg	Neg
S025	Epithelium	11.65	8.69	8.86	8.45	Pos	Pos	Pos
S026	Epithelium	14.94	10.47	10.77	9.95	Pos	Pos	Pos
S027	Epithelium	Neg	Neg	Neg	Neg	Neg	Neg	Neg
S028	Epithelium	Neg	34.54	28.33	28.46	Pos	Neg	Neg
S029	Vesicular fluids	Neg	Neg	Neg	Neg	Neg	Neg	Neg
S030	Epithelium	34.15	23.57	24.68	23.93	Pos	Neg	Pos
S031	Epithelium	19.94	11.06	11.63	10.77	Pos	Neg	Pos
S032	Epithelium	31.91	22.92	23.63	23.085	Pos	Neg	Pos
S033	Epithelium	Neg	24.72	23.95	24.475	Pos	Neg	Pos
S034	Epithelium	Neg	24.16	22.92	Neg	Pos	Neg	Pos
S035	Epithelium	24.83	19.23	19.84	19.27	Pos	Neg	Pos
S036	Epithelium	17.03	12.47	13.10	12.465	Pos	Neg	Pos
S037	Epithelium	18.45	14.98	19.74	14.94	Pos	Neg	Pos
S038	Epithelium	15.57	13.35	18.24	13.82	Pos	Neg	Pos
S039	Epithelium	24.14	23.43	24.22	23.75	Pos	Neg	Pos
S040	Epithelium	30.01	26.53	33.90	27.14	Pos	Neg	Neg

a *Conventional RT-PCR described by Rodríguez et al. (41)*.

*Pos, Positive result; Neg, Negative result*.

Different results were obtained when the samples were assessed using the rRT-PCR single assays (Table 5). The single rRT-PCR assays accomplished on the TgRs separately failed to detect some positive samples. Thus, out of 26 positive samples for FMDV, the single rRT-PCR assays accomplished on TgR1 and TgR3 only detected 21 (80.77%), the assay performed on TgR2 only recognized 23 (88.46%), whereas the assay conducted on TgR4 only detected 25 positive samples (96.15%).

From a total of 40 field samples tested, only 12 samples (30.0%) were positive by VI (Table 5), which were also detected as positive by the mMulti-rRT-PCR system. Thus, the VI and mMulti-rRT-PCR only coincided in 46.15% of the FMDV-positive field samples detected. Five of the 14 samples (S005, S009, S028, S039, and S040) that VI failed to detect, contained from medium to low viral loads, represented by high Ct values in the mMulti-RT-PCR (Ct-value >25) (Table 5). However, the remaining nine samples had high viral loads detected by low Ct values (Table 5). Nonetheless, four samples positive by VI method (S003, S006, S012, and S014) resulted negative for some single rRT-PCR assays when considered as individual assays.

Meanwhile, the conventional RT-PCR detected 19 out 26 positive samples (73.07%) (Table 5), obtaining positive results only for those samples that contained high viral loads (Ct value < 25). Furthermore, the conventional RT-PCR also failed to detect some samples that resulted positive by VI and mMulti-rRT-PCR simultaneously (S003, S014, and S016) (Table 5).

## Discussion

A confirmation of FMDV infection leads to serious limitations in animal trade and the implementation of strict control measures. Hence, the constant need to develop more reliable, precise and accurate diagnostic tools by laboratories on charge of FMDV diagnosis. Even though VI was considered for a long time the gold standard method for case confirmation of FMDV, this method lacks an optimum degree of sensitivity to detect carriers or animals infected with low viral loads (15, 45). For this reason, the rRT-PCR assays have been considered essential tools in the diagnosis of this viral agent [reviewed in Hoffmann et al. (8)]. Nevertheless, the rRT-PCR has among its main drawbacks its ability to produce false positives (11). Therefore, for the detection of viral agents of interest, especially those listed by the OIE, the use of strategies for identifying multiple targets in the genome of the agents (multi-check strategy) is a procedure to be followed by diagnostic (8).

On the other hand, the high variability of the FMDV genome represents a constant challenge for molecular diagnosis of this agent based on nucleic acid detection. Therefore, diagnostic methods based on rRT-PCR previously established need to be checked under the principle of “ongoing evaluation” to redirect possible failures to detect emergent strains (46). The current availability of viral sequences in public databases, together with the development of novel bioinformatic tools, offer the opportunity to check and improve molecular diagnostic method based on nucleic acid detection.

Even though different regions of FMDV genome have been used for the generic detection of this viral agent [reviewed in Hoffmann et al. (8)], the most common targets used for this purpose have been framed into the *IRES* and *3D*^*pol*^ regions (9, 10, 44). In the current work, one TgR (TgR1) within the *IRES* region and three TgRs (TgR2-TgR4) within the *3D*^*pol*^ region were analyzed regarding their variability in different FMDV strains. Although the *IRES* region is highly conserved for most of the viral species (47) the results obtained from entropy plots showed that the TgR included within the *IRES* region showed higher variability values compared to the three TgRs within the *3D*^*Pol*^.

One of the most commonly used formats due to its straightforward design and interpretation is Taqman type assays (11). However, recent reports have showed many escapes of viral strains with high variability to this type of assay (12, 48). These failures are favored by the appearance of nucleotide changes in the sequence of the viral agents, leading to errors in hybridization between the probe and the template (45). In the current study, the *in-silico* analysis revealed several sequences with a high probability of escaping to detection caused by errors in the hybridization between the target sequence and the probe. This fact supports our strategy of developing an assay not only able to detect several targets at the same time but also based on *SYBR-Green I* to increase the capacity of detection of the mMulti-rRT-PCR assay proposed.

*SYBR-Green I* real-time PCRs coupled to melting curves analysis provide a reliable method for the detection and differentiation of nucleic acid targets (49). Moreover, these assays are capable of discriminating between different viral strains based on genomic sequence variability (39). Variations of 1°C in the melting curve resolution values of the amplification products usually are enough to guarantee the differentiation between viral species or strains. Hence, *SYBR-Green I* rPCRs coupled to melting curves have been used in the diagnosis and genotyping of different viral agents (40, 50).

The mMulti-rRT-PCR assay was developed and validated from the conception of a multi-type assay. This format does not allow mixing different primer pairs in a single reaction, but it provides the advantage that different targets are amplified simultaneously in the same carousel, saving time in the emission of diagnostic results (11). When a proper standardization and optimization of the parameters is not rigorously conducted, the rPCR assays can present different challenges including low sensitivity and poor specificity (51). The poor specificity problem needs extreme attention since undesirable products could be amplified more efficiently than the specific target causing a depletion of the reaction components and reducing its efficiency (49). In fact, unoptimized rRT-PCR assays can only achieve amplification efficiency values not exceeding 75%. To avoid these problems the mMulti-rRT-PCR assay proposed here was strictly optimized taking into account all the reaction parameters, that included those involved with both the system (critical components of the PCR reaction) (49) and the environment (thermodynamic parameters) (52). All amplification reactions had an elevated stringency and yield, facilitating the specific amplifications of all targets with high analytical sensitivity and specificity, independently of the size and Tm-value of each amplicon. Even though all amplification efficiency values were elevated, the lowest efficiency value obtained from the TgR1 could be induced by the high level of degeneracy in the primer pairs flanking this region (9), which has been associated with a decrease in the amplification efficiency (53). Nevertheless, the high analytical sensitivity obtained for this amplification reaction allows its use for FMDV diagnosis directly in clinical samples.

The performance of an rRT-PCR assay on clinical field samples obtained from animals affected by the disease is a fundamental step for its use as accurate diagnostic tool (35). In South America, serotype A and O are currently circulating, while serotype C is considered eradicated. The application of the mMulti-rRT-PCR developed in this work on field samples available from Venezuela, allowed the detection of a higher number of FMDV positive samples than when using conventional RT-PCR assay or VI test. This could be caused by several factors. On the one hand, real-time PCR (rPCR), detects the fluorescent signal released by the specific amplification products providing the assay with a higher sensitivity when compared to conventional PCR (37, 52). On the other hand, the fact that VI failed in the detection of FMDV samples containing medium to low viral loads could be associated to the intrinsic limitations of the method. However, VI also failed to detect samples that yielded low Ct-values, which can be correlated with high viral loads; therefore, a successful viral isolation could have been obtained from them. However, the deterioration of the samples by acidification (all samples were confirmed by pH measurement), caused by an incorrect preservation, could have impaired the successful viral isolation, since FMDV is extremely labile in low pH levels. This wrongful practice affects the VI performance but it does not affect the rRT-PCR results. Hence, this could be the cause of the discrepancy between the results from the Multi-rRT-PCR and VI in the samples with high viral loads. It is also important to denote that four positive samples by VI (S003, S006, S012, and S014) resulted negative for some single rRT-PCR assays when these were considered as individual assays, highlighting the importance of using a multiple strategy for the detection of FMDV, especially of new emerging strains.

## Conclusion

In the current study a novel assay is presented as an alternative method allowing a “double check” approach detection, to be used mainly in outbreak scenarios with atypical variant strains circulation of FMDV. From a sequence dataset that included all complete genomes of FMDV available at GenBank Database and bioinformatic approaches, the system was designed to survey multiple regions of the genome simultaneously increasing the probability of pan/FMDV detection. We demonstrate that the assay proposed is a reliable and rapid diagnostic method for sensitive and specific detection of pan/FMDV.

## Author contributions

LP designed the research. LP, LR, and LC performed the *in silico* analyses. LR, LC, and DR performed the molecular biology analyses. AÁ and CP performed the virological analyses. LR, LP, JN, and CP analyzed and interpreted the data. LP, JN, and LR wrote the paper. LG, HD, and JN edit the paper and provided intellectual inputs. All the authors read and approved the final version of the manuscript.

### Conflict of interest statement

The authors declare that the research was conducted in the absence of any commercial or financial relationships that could be construed as a potential conflict of interest.

## References

[1] Jamal SM Belsham GJ. Foot-and-mouth disease: past, present and future. Vet Res. (2013) 44:116. 10.1186/1297-9716-44-116 24308718 PMC4028749

[2] Alexandersen S Quan M Murphy C Knight J Zhang Z. Studies of quantitative parameters of virus excretion and transmission in pigs and cattle experimentally infected with foot-and-mouth disease virus. J Comp Pathol. (2003) 129:268–82. 10.1016/S0021-9975(03)00045-8 14554125

[3] Smith MT Bennett AM Grubman MJ Bundy BC. Foot-and-mouth disease: technical and political challenges to eradication. Vaccine (2014) 32:3902–8. 10.1016/j.vaccine.2014.04.038 24785105

[4] Moreno E Ojosnegros S Garcia-Arriaza J Escarmis C Domingo E Perales C. Exploration of sequence space as the basis of viral RNA genome segmentation. Proc Natl Acad Sci USA. (2014) 111:6678–83. 10.1073/pnas.1323136111 24757055 PMC4020086

[5] Rweyemamu M Roeder P Mackay D Sumption K Brownlie J Leforban Y. Epidemiological patterns of foot-and-mouth disease worldwide. Transbound Merg Dis. (2008) 55:57–72. 10.1111/j.1865-1682.2007.01013.x 18397509

[6] Paton DJ Sumption KJ Charleston B. Options for control of foot-and-mouth disease: knowledge, capability and policy. Philos Transac R Soc Lond B Biol Sci. (2009) 364:2657–67. 10.1098/rstb.2009.0100 19687036 PMC2865093

[7] Stenfeldt C Belsham GJ. Detection of foot-and-mouth disease virus RNA in pharyngeal epithelium biopsy samples obtained from infected cattle: investigation of possible sites of virus replication and persistence. Vet Microbiol. (2012) 154:230–9. 10.1016/j.vetmic.2011.07.007 21831538

[8] Hoffmann B Beer M Reid SM Mertens P Oura CA van Rijn PA. A review of RT-PCR technologies used in veterinary virology and disease control: sensitive and specific diagnosis of five livestock diseases notifiable to the World Organisation for Animal Health. Vet Microbiol. (2009) 139:1–23. 10.1016/j.vetmic.2009.04.034 19497689

[9] Reid SM Ferris NP Hutchings GH Zhang Z Belsham GJ Alexandersen S. Detection of all seven serotypes of foot-and-mouth disease virus by real-time, fluorogenic reverse transcription polymerase chain reaction assay. J Virol Methods (2002) 105:67–80. 10.1016/S0166-0934(02)00081-2 12176143

[10] Callahan JD Brown F Osorio FA Sur JH Kramer E Long GW. Use of a portable real-time reverse transcriptase-polymerase chain reaction assay for rapid detection of foot-and-mouth disease virus. J Am Vet Med Assoc. (2002) 220:1636–42. 10.2460/javma.2002.220.1636 12051502

[11] Belak S. Molecular diagnosis of viral diseases, present trends and future aspects A view from the OIE collaborating centre for the application of polymerase chain reaction methods for diagnosis of viral diseases in veterinary medicine. Vaccine (2007) 25:5444–52. 10.1016/j.vaccine.2006.11.068 17224207 PMC7115665

[12] Tam S Clavijo A Engelhard EK Thurmond MC. Fluorescence-based multiplex real-time RT-PCR arrays for the detection and serotype determination of foot-and-mouth disease virus. J Virol Methods (2009) 161:183–91. 10.1016/j.jviromet.2009.04.033 19427333

[13] McKillen J McMenamy M Reid SM Duffy C Hjertner B King DP. Pan-serotypic detection of foot-and-mouth disease virus using a minor groove binder probe reverse transcription polymerase chain reaction assay. J Virol Methods (2011) 174:117–9. 10.1016/j.jviromet.2011.03.008 21419170

[14] Pierce KE Mistry R Reid SM Bharya S Dukes JP Hartshorn C. Design and optimization of a novel reverse transcription linear-after-the-exponential PCR for the detection of foot-and-mouth disease virus. J Appl Microbiol. (2010) 109:180–9. 10.1111/j.1365-2672.2009.04640.x 20028437

[15] Reid SM Pierce KE Mistry R Bharya S Dukes JP Volpe C. Pan-serotypic detection of foot-and-mouth disease virus by RT linear-after-the-exponential PCR. Mol Cell Probes (2010) 24:250–5. 10.1016/j.mcp.2010.04.004 20433917

[16] Dukes JP King DP Alexandersen S. Novel reverse transcription loop-mediated isothermal amplification for rapid detection of foot-and-mouth disease virus. Arch Virol. (2006) 151:1093–106. 10.1007/s00705-005-0708-5 16453084

[17] Chen HT Zhang J Liu YS Liu XT. Detection of foot-and-mouth disease virus RNA by reverse transcription loop-mediated isothermal amplification. Virol J. (2011) 8:510. 10.1186/1743-422X-8-510 22070774 PMC3222687

[18] Knight-Jones TJ Robinson L Charleston B Rodriguez LL Gay CG Sumption KJ. Global foot-and-mouth disease research update and gap analysis: 4 - diagnostics. Transbound Emerg Dis. (2016) 63(Suppl. 1):42–8. 10.1111/tbed.12523 27320165

[19] Ambagala A Fisher M Goolia M Nfon C Furukawa-Stoffer T Ortega Polo R. Field-deployable reverse transcription-insulated isothermal PCR (RT-iiPCR) assay for rapid and sensitive detection of foot-and-mouth disease virus. Transbound Emerg Dis. (2017) 64:1610–23. 10.1111/tbed.12554 27589902 PMC7169878

[20] Abd El Wahed A El-Deeb A El-Tholoth M Abd El Kader H Ahmed A Hassan S A portable reverse transcription recombinase polymerase amplification assay for rapid detection of foot-and-mouth disease virus. PloS ONE (2013) 8:e71642 10.1371/journal.pone.0071642 23977101 PMC3748043

[21] Sanchez JA Pierce KE Rice JE Wangh LJ. Linear-after-the-exponential (LATE)-PCR: an advanced method of asymmetric PCR and its uses in quantitative real-time analysis. Proc Natl Acad Sci USA. (2004) 101:1933–8. 10.1073/pnas.0305476101 14769930 PMC357030

[22] Pierce KE Sanchez JA Rice JE Wangh LJ. Linear-After-The-Exponential (LATE)-PCR: primer design criteria for high yields of specific single-stranded DNA and improved real-time detection. Proc Natl Acad Sci USA. (2005) 102:8609–14. 10.1073/pnas.0501946102 15937116 PMC1150831

[23] Notomi T Okayama H Masubuchi H Yonekawa T Watanabe K Amino N. Loop-mediated isothermal amplification of DNA. Nucleic Acids Res. (2000) 28:E63. 10.1093/nar/28.12.e63 10871386 PMC102748

[24] Postel A Perez LJ Perera CL Schmeiser S Meyer D Meindl-Boehmer A. Development of a new LAMP assay for the detection of CSFV strains from Cuba: a proof-of-concept study. Arch Virol. (2015) 160:1435–48. 10.1007/s00705-015-2407-1 25877822

[25] Stenfeldt C Lohse L Belsham GJ. The comparative utility of oral swabs and probang samples for detection of foot-and-mouth disease virus infection in cattle and pigs. Vet Microbiol. (2013) 162:330–7. 10.1016/j.vetmic.2012.09.008 23022683

[26] OIE M Chapter 2.7.1: Border disease. In: OIE Manual of Diagnostic Tests and Vaccines. Paris: Office International des Epizooties (2017).

[27] OIE M Chapter 2.1.8: Foot and mouth disease (infection with foot and mouth disease virus). In: OIE Manual of Diagnostic Tests and Vaccines. Paris: Office International des Epizooties (2017).

[28] OIE M Chapter 2.1.23. Vesicular stomatitis. In: OIE Manual of Diagnostic Tests and Vaccines. Paris: Office International des Epizooties (2015).

[29] OIE M Chapter 2.4.7. Bovine viral diarrhoea. In: OIE Manual of Diagnostic Tests and Vaccines. Paris: Office International des Epizooties (2015).

[30] OIE M Chapter 2.8.3. Classical swine fever (Hog colera) In: OIE Manual of Diagnostic Tests and Vaccines. Paris: Office International des Epizooties (2014).

[31] Perez LJ Diaz de Arce H. A RT-PCR assay for the detection of encephalomycarditis virus infections in pigs. Brazil J Microbiol. (2009) 40:988–93. 10.1590/S1517-838220090004000034 24031451 PMC3768574

[32] Diaz de Arce H Perez LJ Frias MT Rosell R Tarradas J Nunez JI A multiplex RT-PCR assay for the rapid and differential diagnosis of classical swine fever and other pestivirus infections. Vet Microb. (2009) 139:245–52. 10.1016/j.vetmic.2009.06.004 19577384

[33] Moniwa M Clavijo A Li M Collignon B Kitching PR. Performance of a foot-and-mouth disease virus reverse transcription-polymerase chain reaction with amplification controls between three real-time instruments. J Vet Diagnos Invest. (2007) 19:9–20. 10.1177/104063870701900103 17459827

[34] Sáiz M De La Morena DB Blanco E Nunez JI Fernandez R Sanchez-Vizcaino JM. Detection of foot-and-mouth disease virus from culture and clinical samples by reverse transcription-PCR coupled to restriction enzyme and sequence analysis. Vet Res. (2003) 34:105–17. 10.1051/vetres:2002059 12588687

[35] Ferris NP King DP Reid SM Hutchings GH Shaw AE Paton DJ. Foot-and-mouth disease virus: a first inter-laboratory comparison trial to evaluate virus isolation and RT-PCR detection methods. Vet Microb. (2006) 117:130–40. 10.1016/j.vetmic.2006.06.001 16846700

[36] Hall TA BioEdit: a user-friendly biological sequence alignment editor and analysis program for Windows 95/98/NT. Nucleic Acids Symp Series (1999) 41:95–8.

[37] Perez LJ Diaz de Arce H Cilloni F Salviato A Marciano S Perera CL. An SYBR Green-based real-time RT-PCR assay for the detection of H5 hemagglutinin subtype avian influenza virus. Mol Cell Probes (2012) 26:137–45. 10.1016/j.mcp.2012.02.001 22421464

[38] Xia X Xie Z. DAMBE: software package for data analysis in molecular biology and evolution. J Heredity (2001) 92:371–3. 10.1093/jhered/92.4.371 11535656

[39] Perez LJ Perera CL Frias MT Nunez JI Ganges L de Arce HD. A multiple SYBR Green I-based real-time PCR system for the simultaneous detection of porcine circovirus type 2, porcine parvovirus, pseudorabies virus and Torque teno sus virus 1 and 2 in pigs. J |Virol Methods (2012) 179:233–41. 10.1016/j.jviromet.2011.11.009 22119629

[40] Acevedo AM Perera CL Vega A Rios L Coronado L Relova D. A duplex SYBR Green I-based real-time RT-PCR assay for the simultaneous detection and differentiation of Massachusetts and non-Massachusetts serotypes of infectious bronchitis virus. Mol Cell Probes (2013) 27:184–92. 10.1016/j.mcp.2013.06.001 23810983

[41] Rodriguez A Nunez JI Nolasco G Ponz F Sobrino F de Blas C. Direct PCR detection of foot-and-mouth disease virus. J Virol Methods (1994) 47:345–9. 8071421 10.1016/0166-0934(94)90030-2

[42] Fronhoffs S Totzke G Stier S Wernert N Rothe M Bruning T. A method for the rapid construction of cRNA standard curves in quantitative real-time reverse transcription polymerase chain reaction. Mol Cell Probes (2002) 16:99–110. 10.1006/mcpr.2002.0405 12030760

[43] Huang S Salituro J Tang N Luk KC Hackett J Jr Swanson P. Thermodynamically modulated partially double-stranded linear DNA probe design for homogeneous real-time PCR. Nucleic acids Res. (2007) 35:e101. 10.1093/nar/gkm551 17693434 PMC2018630

[44] Bolin SR Ridpath JF Black J Macy M Roblin R. Survey of cell lines in the American Type Culture Collection for bovine viral diarrhea virus. J Virol Methods (1994) 48:211–21. 7989438 10.1016/0166-0934(94)90120-1

[45] King DP Ferris NP Shaw AE Reid SM Hutchings GH Giuffre AC. Detection of foot-and-mouth disease virus: comparative diagnostic sensitivity of two independent real-time reverse transcription-polymerase chain reaction assays. J Vet Diagn Invest. (2006) 18:93–7. 10.1177/104063870601800114 16566264

[46] Reid SM Mioulet V Knowles NJ Shirazi N Belsham GJ King DP. Development of tailored real-time RT-PCR assays for the detection and differentiation of serotype O, A and Asia-1 foot-and-mouth disease virus lineages circulating in the Middle East. J Virol Methods (2014) 207:146–53. 10.1016/j.jviromet.2014.07.002 25016065

[47] Komar AA Mazumder B Merrick WC. A new framework for understanding IRES-mediated translation. Gene (2012) 502:75–86. 10.1016/j.gene.2012.04.039 22555019 PMC3361623

[48] Leifer I Everett H Hoffmann B Sosan O Crooke H Beer M. Escape of classical swine fever C-strain vaccine virus from detection by C-strain specific real-time RT-PCR caused by a point mutation in the primer-binding site. J Virol Methods (2010) 166:98–100. 10.1016/j.jviromet.2010.03.004 20332004

[49] Perez LJ Diaz de Arce H Tarradas J Rosell R Perera CL Munoz M. Development and validation of a novel SYBR Green real-time RT-PCR assay for the detection of classical swine fever virus evaluated on different real-time PCR platforms. J Virol Methods (2011) 174:53–9. 10.1016/j.jviromet.2011.03.022 21458490

[50] Zhu Z Fan H Qi X Qi Y Shi Z Wang H. Development and evaluation of a SYBR green-based real time RT-PCR assay for detection of the emerging avian influenza A (H7N9) virus. PloS ONE (2013) 8:e80028. 10.1371/journal.pone.0080028 24278234 PMC3835827

[51] Elnifro EM Ashshi AM Cooper RJ Klapper PE. Multiplex PCR: optimization and application in diagnostic virology. Clin Microbiol Rev. (2000) 13:559–70. 10.1128/CMR.13.4.559-570.2000 11023957 PMC88949

[52] Carrillo C Lu Z Borca MV Vagnozzi A Kutish GF Rock DL. Genetic and phenotypic variation of foot-and-mouth disease virus during serial passages in a natural host. J Virol. (2007) 81:11341–51. 10.1128/JVI.00930-07 17686868 PMC2045514

[53] Wu L Di DW Zhang D Song B Luo P Guo GQ. Frequent problems and their resolutions by using thermal asymmetric interlaced PCR (TAIL-PCR) to clone genes in Arabidopsis T-DNA tagged mutants. Biotechnol Biotechnol Equipment (2015) 29:260–7. 10.1080/13102818.2014.998161 26019639 PMC4433792

