# Supplementary material for: Multi-Target Strategy for Pan/Foot-and-Mouth Disease Virus (FMDV) Detection: A Combination of Sequences Analysis, in Silico Predictions and Laboratory Diagnostic Evaluation
Source: Front Vet Sci. 2018 Jul 12;5:160. doi: 10.3389/fvets.2018.00160 (PMC6052897; doi:10.3389/fvets.2018.00160)
Supplement: Supplementary file 1 [file Data_Sheet_1.DOCX]

**Supplementary material**

**Multi-target strategy for pan/foot and mouth disease virus (FMDV) detection: combining *in silico* assessment with laboratory diagnostic evaluation.**

Liliam Rios^1^, Carmen L. Perera^2^, Liani Coronado^2^, Damarys Relova^2^, Ana M. Álvarez^3^, Llilianne Ganges^4^, Heidy Díaz de Arce^5^, José I. Núñez^6¶^, Lester J. Pérez^7¶*^.

*CORRESPONDENCE: Dr. Lester J. Pérez: [Lester.Perez@dal.ca](mailto:Lester.Perez@dal.ca)

| **Virus** | **Strain/ Viral isolate** | **Source** | **Multi-rRT-PCR** |
| --- | --- | --- | --- |
| FMDV | FMDV/Strain O1/serotype O | Killed Vaccine (C. A. L. A)a | Positive |
| FMDV | FMDV/Strain A24/serotype A | Killed Vaccine (C. A. L. A)a | Positive |
| FMDVϯ | FMDV/serotype C | CENSA, Cuba | Positive |
| FMDVϯ | FMDV/SAT1 | CENSA, Cuba | Positive |
| FMDVϯ | FMDV/SAT2 | CENSA, Cuba | Positive |
| FMDVϯ | FMDV/SAT3 | CENSA, Cuba | Positive |
| FMDVϯ | FMDV/Asia 1 | CENSA, Cuba | Positive |
| FMDV | FMDV/Isolate 21467/serotype A/VEN/2006 | INIA, Venezuela | Positive |
| FMDV | FMDV/Isolate 21469/serotype A/VEN/2006 | INIA, Venezuela | Positive |
| FMDV | FMDV/Isolate 21486/serotype O/VEN/2006 | INIA, Venezuela | Positive |
| FMDV | FMDV/Isolate 21501/serotype A/VEN/2006 | INIA, Venezuela | Positive |
| FMDV | FMDV/Isolate 21513/serotype A/VEN/2007 | INIA, Venezuela | Positive |
| FMDV | FMDV/Isolate 21533/serotype A/VEN/2007 | INIA, Venezuela | Positive |
| FMDV | FMDV/Isolate 21555/serotype O/VEN/2007 | INIA, Venezuela | Positive |
| FMDV | FMDV/Isolate 21557/serotype A/VEN/2007 | INIA, Venezuela | Positive |
| FMDV | FMDV/Isolate 21564/serotype A/VEN/2007 | INIA, Venezuela | Positive |
| FMDV | FMDV/Isolate 21593/serotype A/VEN/2007 | INIA, Venezuela | Positive |
| FMDV | FMDV/Isolate 21598/serotype A/VEN/2007 | INIA, Venezuela | Positive |
| FMDV | FMDV/Isolate 21624/serotype A/VEN/2007 | INIA, Venezuela | Positive |
| FMDV | FMDV/Isolate 21625/serotype A/VEN/2007 | INIA, Venezuela | Positive |
| FMDV | FMDV/Isolate 21627/serotype A/VEN/2007 | INIA, Venezuela | Positive |
| FMDV | FMDV/Isolate 21646/serotype A/VEN/2007 | INIA, Venezuela | Positive |
| FMDV | FMDV/Isolate 21650/serotype A/VEN/2007 | INIA, Venezuela | Positive |
| FMDV | FMDV/Isolate 21652/serotype A/VEN/2007 | INIA, Venezuela | Positive |
| FMDV | FMDV/Isolate 21653/serotype A/VEN/2007 | INIA, Venezuela | Positive |
| FMDV | FMDV/Isolate 21655/serotype A/VEN/2007 | INIA, Venezuela | Positive |
| FMDV | FMDV/Isolate 21661/serotype A/VEN/2007 | INIA, Venezuela | Positive |
| FMDV | FMDV/Isolate 21662/serotype A/VEN/2007 | INIA, Venezuela | Positive |
| FMDV | FMDV/Isolate 21666/serotype O/VEN/2007 | INIA, Venezuela | Positive |
| FMDV | FMDV/Isolate 21672/serotype A/VEN/2007 | INIA, Venezuela | Positive |
| FMDV | FMDV/Isolate 21675/serotype A/VEN/2007 | INIA, Venezuela | Positive |
| FMDV | FMDV/Isolate 21683/serotype O/VEN/2007 | INIA, Venezuela | Positive |
| FMDV | FMDV/Isolate 21684/serotype A/VEN/2007 | INIA, Venezuela | Positive |
| FMDV | FMDV/Isolate 21705/serotype A/VEN/2008 | INIA, Venezuela | Positive |
| FMDV | FMDV/Isolate 21727/serotype A/VEN/2008 | INIA, Venezuela | Positive |
| FMDV | FMDV/Isolate 21735/serotype A/VEN/2008 | INIA, Venezuela | Positive |
| FMDV | FMDV/Isolate 21744/serotype O/VEN/2008 | INIA, Venezuela | Positive |
| FMDV | FMDV/Isolate 21755/serotype A/VEN/2008 | INIA, Venezuela | Positive |

**Table S1.** Viruses and genetic material used in this study

| **Virus** | **Strain/ Viral isolate** | **Source** | **Multi-rRTPCR** |
| --- | --- | --- | --- |
| FMDV | FMDV/Isolate 21759/serotype A/VEN/2008 | INIA, Venezuela | Positive |
| FMDV | FMDV/Isolate 21768/serotype O/VEN/2008 | INIA, Venezuela | Positive |
| FMDV | FMDV/Isolate 21786/serotype A/VEN/2008 | INIA, Venezuela | Positive |
| FMDV | FMDV/Isolate 21789/serotype A/VEN/2008 | INIA, Venezuela | Positive |
| FMDV | FMDV/Isolate 21792/serotype A/VEN/2008 | INIA, Venezuela | Positive |
| FMDV | FMDV/Isolate 21804/serotype A/VEN/2008 | INIA, Venezuela | Positive |
| FMDV | FMDV/Isolate 21807/serotype O/VEN/2008 | INIA, Venezuela | Positive |
| FMDV | FMDV/Isolate 21838/serotype O/VEN/2008 | INIA, Venezuela | Positive |
| FMDV | FMDV/Isolate 21847/serotype O/VEN/2008 | INIA, Venezuela | Positive |
| FMDV | FMDV/Isolate 21877/serotype O/VEN/2009 | INIA, Venezuela | Positive |
| FMDV | FMDV/Isolate 21895/serotype A/VEN/2009 | INIA, Venezuela | Positive |
| FMDV | FMDV/Isolate 21928/serotype O/VEN/2009 | INIA, Venezuela | Positive |
| FMDV | FMDV/Isolate 21951/serotype A/VEN/2010 | INIA, Venezuela | Positive |
| FMDV | FMDV/Isolate 21560A/serotype A/VEN/2010 | INIA, Venezuela | Positive |
| VSV | VSV/Isolate 21539/serotype New Jersey/VEN/2007 | INIA, Venezuela | Negative |
| VSV | VSV/Isolate 21538/serotype Indiana/VEN/2007 | INIA, Venezuela | Negative |
| VSV | VSV/Isolate 21544/serotype Indiana/VEN/2007 | INIA, Venezuela | Negative |
| VSV | VSV/Isolate 21545/serotype Indiana/VEN/2007 | INIA, Venezuela | Negative |
| VSV | VSV/Isolate 21546/serotype Indiana/VEN/2007 | INIA, Venezuela | Negative |
| VSV | VSV/Isolate 21549/serotype New Jersey/VEN/2007 | INIA, Venezuela | Negative |
| VSV | VSV/Isolate 21550/serotype Indiana/VEN/2007 | INIA, Venezuela | Negative |
| VSV | VSV/Isolate 21551/serotype Indiana/VEN/2007 | INIA, Venezuela | Negative |
| VSV | VSV/Isolate 21552/serotype New Jersey/VEN/2007 | INIA, Venezuela | Negative |
| VSV | VSV/Isolate 21576/serotype New Jersey/VEN/2007 | INIA, Venezuela | Negative |
| VSV | VSV/Isolate 21579/serotype New Jersey/VEN/2007 | INIA, Venezuela | Negative |
| VSV | VSV/Isolate 21580/serotype New Jersey/VEN/2007 | INIA, Venezuela | Negative |
| VSV | VSV/Isolate 21582/serotype New Jersey/VEN/2007 | INIA, Venezuela | Negative |
| VSV | VSV/Isolate 21585/serotype New Jersey/VEN/2007 | INIA, Venezuela | Negative |
| VSV | VSV/Isolate 21595/serotype New Jersey/VEN/2007 | INIA, Venezuela | Negative |
| VSV | VSV/Isolate 21601/serotype New Jersey/VEN/2007 | INIA, Venezuela | Negative |
| VSV | VSV/Isolate 21607/serotype New Jersey/VEN/2007 | INIA, Venezuela | Negative |
| VSV | VSV/Isolate 21609/serotype New Jersey/VEN/2007 | INIA, Venezuela | Negative |
| VSV | VSV/Isolate 21612/serotype New Jersey/VEN/2007 | INIA, Venezuela | Negative |
| VSV | VSV/Isolate 21620/serotype New Jersey/VEN/2007 | INIA, Venezuela | Negative |
| VSV | VSV/Isolate 21623/serotype New Jersey/VEN/2007 | INIA, Venezuela | Negative |
| VSV | VSV/Isolate 21628/serotype New Jersey/VEN/2007 | INIA, Venezuela | Negative |
| VSV | VSV/Isolate 21630/serotype New Jersey/VEN/2007 | INIA, Venezuela | Negative |
| VSV | VSV/Isolate 21632/serotype New Jersey/VEN/2007 | INIA, Venezuela | Negative |

**TableS1. Cont.**

| **Virus** | **Strain/ Viral isolate** | **Fuente** | **Multi-rRTPCR** |
| --- | --- | --- | --- |
| VSV | VSV/Isolate 21634/serotype New Jersey/VEN/2007 | INIA, Venezuela | Negative |
| VSV | VSV/Isolate 21639/serotype New Jersey/VEN/2007 | INIA, Venezuela | Negative |
| VSV | VSV/Isolate 21658/serotype New Jersey/VEN/2007 | INIA, Venezuela | Negative |
| VSV | VSV/Isolate 21668/serotype New Jersey/VEN/2007 | INIA, Venezuela | Negative |
| VSV | VSV/Isolate 21673/serotype New Jersey/VEN/2007 | INIA, Venezuela | Negative |
| VSV | VSV/Isolate 21689/serotype New Jersey/VEN/2007 | INIA, Venezuela | Negative |
| VSV | VSV/Isolate 21716/serotype New Jersey/VEN/2008 | INIA, Venezuela | Negative |
| VSV | VSV/Isolate 21720/serotype New Jersey/VEN/2008 | INIA, Venezuela | Negative |
| VSV | VSV/Isolate 21726/serotype New Jersey/VEN/2008 | INIA, Venezuela | Negative |
| VSV | VSV/Isolate 21742/serotype New Jersey/VEN/2008 | INIA, Venezuela | Negative |
| VSV | VSV/Isolate 21747/serotype New Jersey/VEN/2008 | INIA, Venezuela | Negative |
| VSV | VSV/Isolate 21748/serotype New Jersey/VEN/2008 | INIA, Venezuela | Negative |
| VSV | VSV/Isolate 21749/serotype New Jersey/VEN/2008 | INIA, Venezuela | Negative |
| VSV | VSV/Isolate 21750/serotype New Jersey/VEN/2008 | INIA, Venezuela | Negative |
| VSV | VSV/Isolate 21751/serotype New Jersey/VEN/2008 | INIA, Venezuela | Negative |
| VSV | VSV/Isolate 21753/serotype New Jersey/VEN/2008 | INIA, Venezuela | Negative |
| VSV | VSV/Isolate 21754/serotype New Jersey/VEN/2008 | INIA, Venezuela | Negative |
| VSV | VSV/Isolate 21756/serotype New Jersey/VEN/2008 | INIA, Venezuela | Negative |
| VSV | VSV/Isolate 21757/serotype New Jersey/VEN/2008 | INIA, Venezuela | Negative |
| VSV | VSV/Isolate 21760/serotype New Jersey/VEN/2008 | INIA, Venezuela | Negative |
| VSV | VSV/Isolate 21777/serotype New Jersey/VEN/2008 | INIA, Venezuela | Negative |
| VSV | VSV/Isolate 21778/serotype New Jersey/VEN/2008 | INIA, Venezuela | Negative |
| VSV | VSV/Isolate 21784/serotype New Jersey/VEN/2008 | INIA, Venezuela | Negative |
| VSV | VSV/Isolate 21799/serotype New Jersey/VEN/2008 | INIA, Venezuela | Negative |
| VSV | VSV/Isolate 21801/serotype New Jersey/VEN/2008 | INIA, Venezuela | Negative |
| VSV | VSV/Isolate 21801/serotype New Jersey/VEN/2008 | INIA, Venezuela | Negative |
| VSV | VSV/Isolate 21805/serotype New Jersey/VEN/2008 | INIA, Venezuela | Negative |
| VSV | VSV/Isolate 21806/serotype New Jersey/VEN/2008 | INIA, Venezuela | Negative |
| VSV | VSV/Isolate 21814/serotype New Jersey/VEN/2008 | INIA, Venezuela | Negative |
| VSV | VSV/Isolate 21815/serotype New Jersey/VEN/2008 | INIA, Venezuela | Negative |
| VSV | VSV/Isolate 21827/serotype New Jersey/VEN/2008 | INIA, Venezuela | Negative |
| VSV | VSV/Isolate 21832/serotype New Jersey/VEN/2008 | INIA, Venezuela | Negative |
| VSV | VSV/Isolate 21834/serotype New Jersey/VEN/2008 | INIA, Venezuela | Negative |
| VSV | VSV/Isolate 21851/serotype New Jersey/VEN/2008 | INIA, Venezuela | Negative |
| VSV | VSV/Isolate 21857/serotype New Jersey/VEN/2008 | INIA, Venezuela | Negative |
| VSV | VSV/Isolate 21865/serotype New Jersey/VEN/2008 | INIA, Venezuela | Negative |
| VSV | VSV/Isolate 21873/serotype New Jersey/VEN/2009 | INIA, Venezuela | Negative |
| VSV | VSV/Isolate 21883/serotype New Jersey/VEN/2009 | INIA, Venezuela | Negative |

**TableS1. Cont.**

| **Virus** | **Strain/ Viral isolate** | **Fuente** | **Multi-rRTPCR** |
| --- | --- | --- | --- |
| VSV | VSV/Isolate 21898/serotype New Jersey/VEN/2009 | INIA, Venezuela | Negative |
| VSV | VSV/Isolate 21900/serotype Indiana/VEN/2009 | INIA, Venezuela | Negative |
| VSV | VSV/Isolate 21904/serotype New Jersey/VEN/2009 | INIA, Venezuela | Negative |
| VSV | VSV/Isolate 21905/serotype New Jersey/VEN/2009 | INIA, Venezuela | Negative |
| VSV | VSV/Isolate 21908/serotype New Jersey/VEN/2009 | INIA, Venezuela | Negative |
| VSV | VSV/Isolate 21909/serotype New Jersey/VEN/2009 | INIA, Venezuela | Negative |
| VSV | VSV/Isolate 21911/serotype New Jersey/VEN/2009 | INIA, Venezuela | Negative |
| VSV | VSV/Isolate 21912/serotype New Jersey/VEN/2009 | INIA, Venezuela | Negative |
| VSV | VSV/Isolate 21917/serotype New Jersey/VEN/2009 | INIA, Venezuela | Negative |
| VSV | VSV/Isolate 21918/serotype New Jersey/VEN/2009 | INIA, Venezuela | Negative |
| VSV | VSV/Isolate 21919/serotype New Jersey/VEN/2009 | INIA, Venezuela | Negative |
| VSV | VSV/Isolate 21715/serotype New Jersey/VEN/2008 | INIA, Venezuela | Negative |
| VSV | VSV/Isolate 21922/serotype New Jersey/VEN/2009 | INIA, Venezuela | Negative |
| VSV | VSV/Isolate 21931/serotype New Jersey/VEN/2009 | INIA, Venezuela | Negative |
| VSV | VSV/Isolate 21932/serotype New Jersey/VEN/2009 | INIA, Venezuela | Negative |
| VSV | VSV/Isolate 21936/serotype New Jersey/VEN/2009 | INIA, Venezuela | Negative |
| VSV | VSV/Isolate 21942/serotype New Jersey/VEN/2009 | INIA, Venezuela | Negative |
| VSV | VSV/Isolate 21943/serotype New Jersey/VEN/2009 | INIA, Venezuela | Negative |
| VSV | VSV/Isolate 21944/serotype New Jersey/VEN/2009 | INIA, Venezuela | Negative |
| VSV | VSV/Isolate 21965/serotype New Jersey/VEN/2010 | INIA, Venezuela | Negative |
| VSV | VSV/Isolate 21966/serotype New Jersey/VEN/2010 | INIA, Venezuela | Negative |
| VSV | VSV/Isolate 21973/serotype New Jersey/VEN/2010 | INIA, Venezuela | Negative |
| VSV | VSV/Isolate 21974/serotype New Jersey/VEN/2010 | INIA, Venezuela | Negative |
| VSV | VSV/Isolate 21976/serotype New Jersey/VEN/2010 | INIA, Venezuela | Negative |
| VSV | VSV/Isolate 21979/serotype New Jersey/VEN/2010 | INIA, Venezuela | Negative |
| VSV | VSV/Isolate 21980/serotype New Jersey/VEN/2010 | INIA, Venezuela | Negative |
| VSV | VSV/Isolate 21983/serotype New Jersey/VEN/2010 | INIA, Venezuela | Negative |
| VSV | VSV/Isolate 21984/serotype New Jersey/VEN/2010 | INIA, Venezuela | Negative |
| VSV | VSV/Isolate 21993/serotype New Jersey/VEN/2010 | INIA, Venezuela | Negative |
| VSV | VSV/Isolate 21997/serotype New Jersey/VEN/2010 | INIA, Venezuela | Negative |
| VSV | VSV/Isolate 21585A/serotype New Jersey/VEN/2010 | INIA, Venezuela | Negative |
| VSV | VSV/Isolate 21714A/serotype New Jersey/VEN/2010 | INIA, Venezuela | Negative |
| VSV | VSV/Isolate 21754A/serotype New Jersey/VEN/2010 | INIA, Venezuela | Negative |
| VSV | VSV/Isolate 21777A/serotype New Jersey/VEN/2010 | INIA, Venezuela | Negative |
| VSV | VSV/Isolate 21992A/serotype New Jersey/VEN/2010 | INIA, Venezuela | Negative |

**TableS1. Cont.**

| **Virus** | **Cepa de referencia/ Aislado viral** | **Fuente** | **Multi-rRTPCR** |
| --- | --- | --- | --- |
| CSFV | Margarita/Genotype 1 | CENSA, Cuba | Negative |
| CSFV | Paderbor/Genotype 2 | CENSA, Cuba | Negative |
| CSFV | Congenital Tremor/Genotype 3 | CENSA, Cuba | Negative |
| BVDV1 | NADL/subtype 1a | CENSA, Cuba | Negative |
| BVDV2 | New York | EU Reference Laboratory for CSF, Germany | Negative |
| BDV | Moredum/Genotype 1 | EU Reference Laboratory for CSF, Germany | Negative |
| BDV | Gifhorn/Genotype 1 | EU Reference Laboratory for CSF, Germany | Negative |
| EMCVa |  | CENSA, Cuba | Negative |
| SVDVϯ |  | CENSA, Cuba | Negative |

**TableS1. Cont.**

**TableS2.** Results of priming efficiency (P.E.) values of each primer pair on the FMDV sequences (P.E. for the primer against a linked sequence/maximal theoretical P.E for a perfect match).

| **GenBank assesion number\| Strain or isolate** | **Primer pair** | | | |
| --- | --- | --- | --- | --- |
|  | **FP/RP-TgR1** | **FP/RP-TgR2** | **FP/RP-TgR3** | **FP/RP-TgR4** |
| AF540910.1\| FMDV SAT 2 clone ZIM/7/83 | (458/458)/(469/469) | (474/474)/(494/494) | (454/454)/(587/587) | (363/492)/(387/440) |
| EF175732.1\| FMDV - type O isolate WFL | (458/458)/(469/469) | (474/474)/(482/482) | (454/454)/(583/583) | (492/492)/(474/474) |
| AY390432.1\| FMDV Asia1 strain YNBS/58 | (458/458)/(469/469) | (474/474)/(482/482) | (454/454)/(587/587) | (487/487)/(444/444) |
| AY593837.1\| FMDV O isolate ouruguay-51 iso51 | (458/458)/(469/469) | (474/474)/(482/482) | (454/454)/(587/587) | (487/487)/(444/444) |
| AY593836.1\| FMDV O isolate ouk2001x iso84 | (458/458)/(469/469) | (474/474)/(482/482) | (454/454)/(587/587) | (487/487)/(516/516) |
| AY593835.1\| FMDV O isolate otaiwan97 iso106/112 | (458/458)/(469/469) | (474/474)/(482/482) | (454/454)/(583/583) | (504/504)/(474/474) |
| AY593834.1\| FMDV O isolate orey-iran iso53 | (458/458)/(312/469) | (474/474)/(482/482) | (454/454)/(409/587) | (362/487)/(516/516) |
| AY593833.1\| FMDV O isolate openghu iso108 | (458/458)/(469/469) | (474/474)/(482/482) | (442/454)/(583/583) | (504/504)/(442/468) |
| AY593832.1\| FMDV O isolate O UK2001-FB | (458/458)/(469/469) | (474/474)/(482/482) | (454/454)/(587/587) | (487/487)/(516/516) |
| AY593831.1\| FMDV O isolate O UK2001-ED | (458/458)/(469/469) | (474/474)/(482/482) | (454/454)/(587/587) | (487/487)/(516/516) |
| AY593830.1\| FMDV O isolate o7poland iso49 | (458/458)/(469/469) | (474/474)/(482/482) | (454/454)/(587/587) | (487/487)/(466/466) |
| AY593829.1\| FMDV O isolate o6pirbright iso58 | (458/458)/(469/469) | (474/474)/(482/482) | (153/454)/(587/587) | (464/487)/(516/516) |
| AY593828.1\| FMDV O isolate o5india iso34 | (458/458)/(469/469) | (474/474)/(482/482) | (454/454)/(587/587) | (487/487)/(516/516) |
| AY593827.1\| FMDV O isolate o3venezuela iso15 | (458/458)/(469/469) | (474/474)/(348/482) | (306/454)/(587/587) | (487/487)/(516/516) |
| AY593826.1\| FMDV O isolate o2brescia iso17 | (458/458)/(469/469) | (474/474)/(482/482) | (454/454)/(587/587) | (487/487)/(516/516) |
| AY593825.1\| FMDV O isolate o1valle iso64 | (458/458)/(255/469) | (474/474)/(442/482) | (454/454)/(587/587) | (439/487)/(441/516) |
| AY593824.1\| FMDV O isolate o1skr iso85 | (458/458)/(469/469) | (474/474)/(482/482) | (454/454)/(587/587) | (487/487)/(516/516) |
| AY593823.1\| FMDV O isolate o1manisa iso87 | (458/458)/(469/469) | (474/474)/(482/482) | (454/454)/(587/587) | (487/487)/(516/516) |
| AY593822.1\| FMDV O isolate o1m11 iso57 | (458/458)/(469/469) | (474/474)/(348/482) | (306/454)/(587/587) | (487/487)/(516/516) |
| AY593821.1\| FMDV O isolate o1caseros iso35 | (458/458)/(469/469) | (474/474)/(482/482) | (454/454)/(587/587) | (469/479)/(466/466) |
| AY593820.1\| FMDV O isolate o1canefa iso59 | (458/458)/(469/469) | (474/474)/(482/482) | (454/454)/(587/587) | (487/487)/(466/466) |
| AY593819.1\| FMDV O isolate o1campos94 iso94 | (458/458)/(469/469) | (474/474)/(482/482) | (454/454)/(587/587) | (487/487)/(466/466) |
| AY593818.1\| FMDV O isolate o1campos iso96 | (458/458)/(469/469) | (474/474)/(482/482) | (454/454)/(587/587) | (487/487)/(466/466) |
| AY593817.1\| FMDV O isolate o1brugge iso79 | (458/458)/(469/469) | (474/474)/(482/482) | (454/454)/(587/587) | (487/487)/(466/466) |
| AY593816.1\| FMDV O isolate o1bfs46 iso46 | (458/458)/(469/469) | (474/474)/(482/482) | (454/454)/(587/587) | (487/487)/(466/466) |
| AY593815.1\| FMDV O isolate o1bfs iso18 | (458/458)/(469/469) | (474/474)/(482/482) | (454/454)/(587/587) | (487/487)/(466/466) |
| AY593814.1\| FMDV O isolate o1argentina iso5 | (458/458)/(469/469) | (474/474)/(482/482) | (454/454)/(587/587) | (487/487)/(466/466) |
| AY593813.1\| FMDV O isolate o11indonesia iso52 | (458/458)/(469/469) | (474/474)/(482/482) | (454/454)/(587/587) | (437/487)/(437/437) |
| AY593812.1\| FMDV O isolate o10phil76 iso76 | (458/458)/(329/469) | (474/474)/(482/482) | (407/454)/(587/587) | (496/496)/(516/516) |
| AY593811.1\| FMDV O isolate o10phil54 iso54 | (458/458)/(329/469) | (474/474)/(482/482) | (407/454)/(587/587) | (496/496)/(516/516) |
| AY593803.1\| FMDV A isolate avenceslau iso70 | (458/458)/(469/469) | (474/474)/(482/482) | (454/454)/(587/587) | (365/487)/(457/457) |
| AY593802.1\| FMDV A isolate A uruguay 2001 iso98 | (458/458)/(331/469) | (474/474)/(482/482) | (454/454)/(587/587) | (471/471)/(466/466) |
| AY593801.1\| FMDV A isolate A30 Uruguay/68 iso90 | (458/458)/(331/469) | (474/474)/(482/482) | (454/454)/(587/587) | (471/471)/(466/466) |
| AY593800.1\| FMDV Asia 1 isolate asia1leb83 iso28 | (458/458)/(469/469) | (474/474)/(482/482) | (454/454)/(587/587) | (487/487)/(516/516) |
| AY593799.1\| FMDV Asia 1 isolate asia1leb4 iso4 | (458/458)/(469/469) | (474/474)/(482/482) | (454/454)/(587/587) | (487/487)/(516/516) |
| AY593798.1\| FMDV Asia 1 isolate asia1leb-89 iso89 | (458/458)/(469/469) | (474/474)/(482/482) | (454/454)/(587/587) | (487/487)/(516/516) |
| AY593797.1\| FMDV Asia 1 isolate asia1-3kimron iso61 | (458/458)/(469/469) | (474/474)/(482/482) | (454/454)/(587/587) | (487/487)/(445/516) |
| AY593796.1\| FMDV Asia 1 isolate asia1-2isrl3-63 iso6 | (458/458)/(469/469) | (474/474)/(315/482) | (454/454)/(473/587) | (496/496)/(445/516) |
| AY593795.1\| FMDV Asia 1 isolate asia1-1pak iso3 | (458/458)/(415/469) | (474/474)/(482/482) | (454/454)/(587/587) | (487/487)/(516/516) |
| AY593794.1\| FMDV A isolate asabana iso68 | (392/458)/(469/469) | (460/460)/(482/482) | (454/454)/(587/587) | (388/479)/(516/516) |
| AY593793.1\| FMDV A isolate aphilippines iso50 | (458/458)/(392/469) | (474/474)/(482/482) | (454/454)/(371/587) | (413/492)/(461/461) |
| AY593792.1\| FMDV A isolate aparma iso55 | (392/458)/(469/469) | (474/474)/(397/482) | (454/454)/(587/587) | (438/479)/(466/466) |
| AY593791.1\| FMDV A isolate airan iso105 | (458/458)/(469/469) | (474/474)/(482/482) | (454/454)/(587/587) | (487/487)/(516/516) |
| AY593790.1\| FMDV A isolate a general lopez iso102 | (458/458)/(331/469) | (474/474)/(482/482) | (454/454)/(587/587) | (471/471)/(466/466) |
| AY593789.1\| FMDV A isolate acanefa iso48 | (458/458)/(288/469) | (474/474)/(410/494) | (454/454)/(587/587) | (496/496)/(253/466) |
| AY593788.1\| FMDV A isolate abrazil iso67 | (458/458)/(181/469) | (474/474)/(482/482) | (454/454)/(587/587) | (385/479)/(440/440) |
| AY593787.1\| FMDV A isolate abage iso63 | (458/458)/(181/469) | (474/474)/(482/482) | (454/454)/(587/587) | (385/479)/(457/457) |
| AY593786.1\| FMDV A isolate aarg Trenquelauquen iso103 | (458/458)/(288/469) | (474/474)/(482/482) | (454/454)/(587/587) | (471/471)/(466/466) |
| AY593785.1\| FMDV A isolate aargp64 iso100 | (458/458)/(331/469) | (474/474)/(482/482) | (454/454)/(587/587) | (471/471)/(466/466) |
| AY593784.1\| FMDV A isolate aargp55 iso99 | (458/458)/(331/469) | (474/474)/(482/482) | (454/454)/(587/587) | (471/471)/(466/466) |
| AY593783.1\| FMDV A isolate aarg2001 iso93 | (458/458)/(331/469) | (474/474)/(482/482) | (454/454)/(587/587) | (471/471)/(466/466) |
| AY593782.1\| FMDV A isolate a argentina 2000 iso104 | (458/458)/(469/469) | (474/474)/(426/482) | (332/454)/(587/587) | (479/479)/(253/466) |
| AY593781.1\| FMDV A isolate a5westerwald iso73 | (458/458)/(469/469) | (474/474)/(455/482) | (454/454)/(587/587) | (438/479)/(466/466) |
| AY593780.1\| FMDV A isolate a5allier iso45 | (458/458)/(469/469) | (474/474)/(392/482) | (454/454)/(587/587) | (438/479)/(253/466) |
| AY593779.1\| FMDV A isolate a4wg iso72 | (458/458)/(469/469) | (474/474)/(482/482) | (454/454)/(587/587) | (487/487)/(516/516) |
| AY593778.1\| FMDV A isolate a4spain iso62 | (432/458)/(469/469) | (449/474)/(455/482) | (454/454)/(587/587) | (438/479)/(516/516) |
| AY593777.1\| FMDV A isolate a4 W Germany iso42 | (458/458)/(469/469) | (474/474)/(482/482) | (454/454)/(587/587) | (487/487)/(516/516) |
| AY593776.1\| FMDV A isolate a3mecklenburg iso81 | (458/458)/(392/469) | (474/474)/(482/482) | (146/454)/(587/587) | (496/496)/(516/516) |
| AY593775.1\| FMDV A isolate a32ven iso36 | (458/458)/(469/469) | (474/474)/(482/482) | (454/454)/(587/587) | (487/487)/(466/466) |
| AY593774.1\| FMDV A isolate a2spain iso7 | (458/458)/(469/469) | (474/474)/(482/482) | (454/454)/(587/587) | (487/487)/(516/516) |
| AY593773.1\| FMDV A isolate a29peru iso37 | (458/458)/(469/469) | (474/474)/(482/482) | (454/454)/(587/587) | (487/487)/(466/466) |
| AY593772.1\| FMDV A isolate a28 Turkey iso44 | (458/458)/(469/469) | (474/474)/(482/482) | (454/454)/(587/587) | (422/487)/(466/466) |
| AY593771.1\| FMDV A isolate a27columbia iso78 | (458/458)/(469/469) | (474/474)/(482/482) | (454/454)/(587/587) | (388/479)/(471/516) |
| AY593770.1\| FMDV A isolate a26arg iso74 | (458/458)/(469/469) | (474/474)/(482/482) | (330/454)/(587/587) | (479/479)/(516/516) |
| AY593769.1\| FMDV A isolate a25 argentina iso38 | (458/458)/(288/469) | (474/474)/(410/494) | (454/454)/(587/587) | (496/496)/(253/466) |
| AY593768.1\| FMDV A isolate a24cruzeiro iso71 | (458/458)/(469/469) | (474/474)/(482/482) | (454/454)/(587/587) | (438/479)/(461/461) |
| AY593767.1\| FMDV A isolate a24 argentina iso9 | (458/458)/(469/469) | (474/474)/(482/482) | (454/454)/(587/587) | (323/479)/(466/466) |
| AY593766.1\| FMDV A isolate a23kenya iso8 | (458/458)/(415/469) | (474/474)/(482/482) | (176/454)/(587/587) | (447/487)/(466/466) |
| AY593765.1\| FMDV A isolate a22turkey iso66 | (458/458)/(469/469) | (474/474)/(482/482) | (454/454)/(587/587) | (487/487)/(466/466) |
| AY593764.1\| FMDV A isolate a22iraq70 iso92 | (458/458)/(415/469) | (474/474)/(397/482) | (454/454)/(587/587) | (487/487)/(444/444) |
| AY593763.1\| FMDV A isolate a22iraq64 iso86 | (458/458)/(415/469) | (474/474)/(397/482) | (454/454)/(587/587) | (487/487)/(444/444) |
| AY593762.1\| FMDV A isolate a22iraq-95 iso95 | (458/458)/(415/469) | (474/474)/(397/482) | (454/454)/(587/587) | (487/487)/(444/444) |
| AY593761.1\| FMDV A isolate a21kenya iso77 | (458/458)/(469/469) | (474/474)/(482/482) | (362/454)/(587/587) | (370/492)/(416/416) |
| AY593760.1\| FMDV A isolate a20ussr iso10 | (458/458)/(469/469) | (474/474)/(482/482) | (454/454)/(587/587) | (438/479)/(253/466) |
| AY593759.1\| FMDV A isolate a1bayern iso41 | (458/458)/(331/469) | (474/474)/(482/482) | (153/454)/(587/587) | (487/487)/(516/516) |
| AY593758.1\| FMDV A isolate a18zulia iso40 | (458/458)/(469/469) | (474/474)/(482/482) | (454/454)/(587/587) | (447/487)/(466/466) |
| AY593757.1\| FMDV A isolate a17 Aguarulbos iso83 | (458/458)/(469/469) | (474/474)/(482/482) | (454/454)/(587/587) | (438/479)/(466/466) |
| AY593756.1\| FMDV A isolate a16belem iso80 | (458/458)/(469/469) | (474/474)/(482/482) | (454/454)/(587/587) | (394/475)/(516/516) |
| AY593755.1\| FMDV A isolate a15thailand iso43 | (458/458)/(469/469) | (474/474)/(482/482) | (454/454)/(587/587) | (487/487)/(516/516) |
| AY593754.1\| FMDV A isolate a14 spain iso39 | (432/458)/(469/469) | (449/474)/(455/482) | (454/454)/(587/587) | (438/479)/(516/516) |
| AY593753.1\| FMDV A isolate a13brazil iso75 | (458/458)/(469/469) | (474/474)/(482/482) | (454/454)/(587/587) | (447/487)/(466/466) |
| AY593752.1\| FMDV A isolate a12valle 119 iso20 | (458/458)/(469/469) | (474/474)/(482/482) | (454/454)/(587/587) | (496/496)/(516/516) |
| AY593751.1\| FMDV A isolate a10holland iso82 | (458/458)/(469/469) | (474/474)/(482/482) | (454/454)/(587/587) | (487/487)/(507/507) |
| AY687334.1\| FMDV - type Asia 1 strain IND 491/97 | (458/458)/(469/469) | (474/474)/(482/482) | (454/454)/(463/587) | (487/487)/(466/466) |
| AY687333.1\| FMDV - type Asia 1 isolate IND 321/01 | (458/458)/(469/469) | (474/474)/(482/482) | (430/454)/(587/587) | (487/487)/(516/516) |
| AF506822.2\| FMDV O strain China/1/99(Tibet) | (458/458)/(469/469) | (474/474)/(482/482) | (454/454)/(587/587) | (487/487)/(516/516) |
| AY593853.1\| FMDV SAT 3 isolate sat3-4bech iso23 | (458/458)/(415/469) | (474/474)/(494/494) | (454/454)/(587/587) | (363/492)/(457/457) |
| AY593852.1\| FMDV SAT 3 isolate sat3-3kenya iso22 | (392/458)/(255/469) | (460/460)/(494/494) | (454/454)/(587/587) | (363/492)/(457/457) |
| AY593851.1\| FMDV SAT 3 isolate sat3-3bech iso29 | (392/458)/(255/469) | (460/460)/(494/494) | (454/454)/(587/587) | (363/492)/(457/457) |
| AY593850.1\| FMDV SAT 3 isolate sat3-2sa iso27 | (432/458)/(415/469) | (449/474)/(410/494) | (454/454)/(587/587) | (352/504)/(440/440) |
| AY593849.1\| FMDV SAT 2 isolate sat2-3kenya-21 | (458/458)/(469/469) | (474/474)/(482/482) | (454/454)/(587/587) | (322/487)/(466/466) |
| AY593848.1\| FMDV SAT 2 isolate sat2-2 iso25 | (458/458)/(469/469) | (474/474)/(494/494) | (454/454)/(371/587) | (328/492)/(440/440) |
| AY593847.1\| FMDV SAT 2 isolate sat2-1rhod iso26 | (392/458)/(469/469) | (460/460)/(410/494) | (454/454)/(587/587) | (367/501)/(440/440) |
| AY593846.1\| FMDV SAT 1 isolate sat1rhod iso33 | (458/458)/(469/469) | (474/474)/(494/494) | (454/454)/(587/587) | (363/492)/(457/457) |
| AY593845.1\| FMDV SAT 1 isolate sat1bot iso47 | (458/458)/(469/469) | (474/474)/(410/494) | (454/454)/(587/587) | (363/492)/(457/457) |
| AY593844.1\| FMDV SAT 1 isolate sat1-7isrl iso12 | (458/458)/(469/469) | (474/474)/(482/482) | (419/454)/(587/587) | (360/487)/(440/440) |
| AY593843.1\| FMDV SAT 1 isolate sat1-6swa iso16 | (458/458)/(200/469) | (474/474)/(410/494) | (454/454)/(587/587) | (363/492)/(457/457) |
| AY593842.1\| FMDV SAT 1 isolate sat1-5sa iso13 | (458/458)/(469/469) | (474/474)/(410/494) | (454/454)/(587/587) | (363/492)/(457/457) |
| AY593841.1\| FMDV SAT 1 isolate sat1-4srhod iso24 | (458/458)/(469/469) | (474/474)/(410/494) | (454/454)/(587/587) | (363/492)/(440/440) |
| AY593840.1\| FMDV SAT 1 isolate sat1-3swa iso14 | (458/458)/(469/469) | (474/474)/(494/494) | (454/454)/(587/587) | (363/492)/(440/440) |
| AY593839.1\| FMDV SAT 1 isolate sat1-20 iso11 | (178/458)/(469/469) | (326/474)/(494/494) | (454/454)/(371/587) | (363/492)/(416/416) |
| AY593838.1\| FMDV SAT 1 isolate sat1-1bech iso30 | (458/458)/(469/469) | (474/474)/(306/494) | (454/454)/(587/587) | (363/492)/(457/457) |
| AY593810.1\| FMDV C isolate cwald iso32 | (392/458)/(469/469) | (460/460)/(482/482) | (454/454)/(587/587) | (436/496)/(446/516) |
| DQ404180.1\| FMDV - type O strain UKG/11/2001 | (458/458)/(469/469) | (474/474)/(482/482) | (454/454)/(587/587) | (487/487)/(516/516) |
| DQ404179.1\| FMDV - type O strain UKG/126/2001 | (458/458)/(469/469) | (474/474)/(482/482) | (454/454)/(587/587) | (487/487)/(516/516) |
| DQ404178.1\| FMDV - type O strain UKG/127/2001 | (458/458)/(469/469) | (474/474)/(482/482) | (454/454)/(587/587) | (487/487)/(516/516) |
| DQ404177.1\| FMDV - type O strain UKG/128/2001 | (458/458)/(469/469) | (474/474)/(482/482) | (454/454)/(587/587) | (487/487)/(516/516) |
| DQ404176.1\| FMDV - type O strain UKG/150/2001 | (458/458)/(469/469) | (474/474)/(482/482) | (454/454)/(587/587) | (487/487)/(516/516) |
| DQ404175.1\| FMDV - type O strain UKG/173/2001 | (458/458)/(469/469) | (474/474)/(482/482) | (454/454)/(587/587) | (487/487)/(516/516) |
| DQ404174.1\| FMDV - type O strain UKG/438/2001 | (458/458)/(469/469) | (474/474)/(482/482) | (454/454)/(587/587) | (487/487)/(516/516) |
| DQ404173.1\| FMDV - type O strain UKG/220/2001 | (458/458)/(469/469) | (474/474)/(482/482) | (454/454)/(587/587) | (487/487)/(516/516) |
| DQ404172.1\| FMDV - type O strain UKG/621/2001 | (458/458)/(469/469) | (474/474)/(482/482) | (454/454)/(587/587) | (487/487)/(516/516) |
| DQ404171.1\| FMDV - type O strain UKG/4569/2001 | (458/458)/(469/469) | (474/474)/(482/482) | (454/454)/(587/587) | (487/487)/(516/516) |
| DQ404169.1\| FMDV - type O strain UKG/7038/2001 | (458/458)/(469/469) | (474/474)/(482/482) | (454/454)/(587/587) | (487/487)/(516/516) |
| DQ404170.1\| FMDV - type O strain UKG/7675/2001 | (458/458)/(469/469) | (474/474)/(482/482) | (454/454)/(587/587) | (487/487)/(516/516) |
| DQ404168.1\| FMDV - type O strain UKG/9011/2001 | (458/458)/(469/469) | (474/474)/(482/482) | (454/454)/(587/587) | (487/487)/(516/516) |
| DQ404167.1\| FMDV - type O strain UKG/9327/2001 | (458/458)/(469/469) | (474/474)/(482/482) | (454/454)/(587/587) | (487/487)/(516/516) |
| DQ404166.1\| FMDV - type O strain UKG/9788/2001 | (458/458)/(469/469) | (474/474)/(482/482) | (454/454)/(587/587) | (487/487)/(516/516) |
| DQ404165.1\| FMDV - type O strain UKG/9964/2001 | (458/458)/(469/469) | (474/474)/(482/482) | (454/454)/(587/587) | (487/487)/(516/516) |
| DQ404164.1\| FMDV - type O strain UKG/11676/2001 | (458/458)/(469/469) | (474/474)/(482/482) | (153/454)/(587/587) | (487/487)/(516/516) |
| DQ404163.1\| FMDV - type O strain UKG/14339/2001 | (458/458)/(469/469) | (474/474)/(482/482) | (454/454)/(587/587) | (487/487)/(516/516) |
| DQ404162.1\| FMDV - type O strain UKG/14476/2001 | (458/458)/(469/469) | (474/474)/(482/482) | (454/454)/(587/587) | (487/487)/(516/516) |
| DQ404161.1\| FMDV - type O strain UKG/14391/2001 | (458/458)/(469/469) | (474/474)/(482/482) | (454/454)/(587/587) | (487/487)/(516/516) |
| DQ404160.1\| FMDV - type O strain UKG/14524/2001 | (458/458)/(469/469) | (474/474)/(482/482) | (454/454)/(587/587) | (487/487)/(516/516) |
| DQ404159.1\| FMDV - type O strain UKG/14603/2001 | (458/458)/(469/469) | (474/474)/(482/482) | (454/454)/(587/587) | (487/487)/(516/516) |
| DQ404158.1\| FMDV - type O strain UKG/15101/2001 | (458/458)/(469/469) | (474/474)/(482/482) | (454/454)/(587/587) | (487/487)/(516/516) |
| DQ478937.1\| FMDV - type O | (458/458)/(331/469) | (474/474)/(482/482) | (381/454)/(371/587) | (444/496)/(354/465) |
| DQ478936.1\| FMDV - type O | (458/458)/(331/469) | (474/474)/(482/482) | (381/454)/(371/587) | (444/496)/(465/465) |
| AY333431.1\| FMDV O isolate O/NY00 | (458/458)/(469/469) | (474/474)/(482/482) | (454/454)/(587/587) | (487/487)/(516/516) |
| AF274010.1\| FMDV C strain C-S8 clone MARLS | (458/458)/(469/469) | (474/474)/(494/494) | (454/454)/(587/587) | (359/479)/(516/516) |
| DQ248888.1\| FMDV - type O isolate lz | (458/458)/(469/469) | (474/474)/(482/482) | (454/454)/(583/583) | (492/492)/(474/474) |
| AF308157.1\| FMDV | (458/458)/(469/469) | (474/474)/(482/482) | (454/454)/(583/583) | (504/504)/(474/474) |
| AF026168.2\| FMDV O strain Chu-Pei | (458/458)/(469/469) | (474/474)/(482/482) | (454/454)/(583/583) | (504/504)/(474/474) |
| AY317098.1\| FMDV HKN/2002 | (458/458)/(469/469) | (474/474)/(482/482) | (454/454)/(583/583) | (442/492)/(474/474) |
| AY304994.1\| FMDV Asia 1 IND 63/72 | (458/458)/(469/469) | (474/474)/(482/482) | (454/454)/(587/587) | (487/487)/(516/516) |
| AF377945.1\| FMDV O/SKR/2000 | (458/458)/(469/469) | (474/474)/(482/482) | (454/454)/(587/587) | (487/487)/(516/516) |
| AY593809.1\| FMDV C5 isolate c5arg iso60 | (458/458)/(469/469) | (474/474)/(482/482) | (290/454)/(587/587) | (416/492)/(516/516) |
| AY593808.1\| FMDV C4 isolate C4 Tierra del Fuego iso2 | (458/458)/(469/469) | (474/474)/(494/494) | (454/454)/(587/587) | (438/479)/(516/516) |
| AY593807.1\| FMDV C3 isolate c3resende iso1 | (458/458)/(469/469) | (474/474)/(482/482) | (454/454)/(587/587) | (492/492)/(516/516) |
| AY593806.1\| FMDV C3 isolate c3ind iso19 | (458/458)/(469/469) | (474/474)/(482/482) | (454/454)/(587/587) | (403/479)/(466/466) |
| AY593805.1\| FMDV C1 isolate c1ober iso88 | (458/458)/(469/469) | (474/474)/(494/494) | (454/454)/(587/587) | (359/479)/(516/516) |
| AY593804.1\| FMDV C1 isolate c1noville iso56 | (458/458)/(469/469) | (474/474)/(494/494) | (454/454)/(587/587) | (359/479)/(516/516) |
| EF117837.1\| FMDV - type A isolate Lindholm 1.3,PAK3/2006 | (458/458)/(469/469) | (474/474)/(482/482) | (454/454)/(587/587) | (409/487)/(466/466) |
| DQ409191.1\| FMDV - type C isolate C-S8p460p5d | (458/458)/(469/469) | (474/474)/(494/494) | (454/454)/(587/587) | (323/479)/(516/516) |
| DQ409190.1\| FMDV - type C isolate C-S8p460d951 | (458/458)/(469/469) | (474/474)/(494/494) | (454/454)/(587/587) | (323/479)/(516/516) |
| DQ409189.1\| FMDV - type C isolate C-S8p460d417 | (458/458)/(469/469) | (474/474)/(494/494) | (454/454)/(587/587) | (323/479)/(516/516) |
| DQ409188.1\| FMDV - type C isolate C-S8p360p5d | (458/458)/(469/469) | (474/474)/(494/494) | (454/454)/(587/587) | (323/479)/(516/516) |
| DQ409187.1\| FMDV - type C isolate C-S8p360d951 | (458/458)/(469/469) | (474/474)/(494/494) | (454/454)/(587/587) | (323/479)/(516/516) |
| DQ409186.1\| FMDV - type C isolate C-S8p360d417 | (458/458)/(469/469) | (474/474)/(494/494) | (454/454)/(587/587) | (323/479)/(516/516) |
| DQ409185.1\| FMDV - type C isolate C-S8p260p3d | (458/458)/(469/469) | (474/474)/(494/494) | (454/454)/(587/587) | (323/479)/(516/516) |
| DQ409184.1\| FMDV - type C isolate C-S8p260d999 | (458/458)/(469/469) | (474/474)/(494/494) | (454/454)/(587/587) | (323/479)/(516/516) |
| DQ409183.1\| FMDV - type C isolate C-S8p260d417 | (458/458)/(469/469) | (474/474)/(494/494) | (454/454)/(587/587) | (323/479)/(516/516) |
| AY359854.1\| FMDV O strain OMIII | (458/458)/(331/469) | (474/474)/(482/482) | (381/454)/(371/587) | (487/487)/(477/516) |
| AJ539141.1\| FMDV O, strain UKG/35/2001 | (458/458)/(469/469) | (474/474)/(482/482) | (454/454)/(587/587) | (487/487)/(516/516) |
| AJ539140.1\| FMDV O, strain SAR/19/2000 | (458/458)/(469/469) | (474/474)/(482/482) | (454/454)/(587/587) | (487/487)/(516/516) |
| AJ539139.1\| FMDV O, strain SKR/2000 | (458/458)/(469/469) | (474/474)/(482/482) | (454/454)/(587/587) | (487/487)/(516/516) |
| AJ539138.1\| FMDV O, strain Tibet/CHA/99 | (458/458)/(469/469) | (474/474)/(482/482) | (454/454)/(587/587) | (487/487)/(516/516) |
| AJ539137.1\| FMDV O, strain TAW/2/99 BOV | (458/458)/(469/469) | (474/474)/(482/482) | (454/454)/(587/587) | (487/487)/(516/516) |
| AJ539136.1\| FMDV O, strain TAW/2/99 TC | (458/458)/(469/469) | (474/474)/(482/482) | (454/454)/(587/587) | (487/487)/(516/516) |
| AJ320488.1\| FMDV O genomic RNA, isolate O1Campos | (458/458)/(469/469) | (474/474)/(482/482) | (454/454)/(587/587) | (487/487)/(466/466) |
| AJ633821.1\| FMDV serotype O, isolate FRA/1/2001 | (458/458)/(469/469) | (474/474)/(482/482) | (454/454)/(587/587) | (487/487)/(516/516) |
| AM409325.1\| FMDV - type C isolate H595 | (458/458)/(469/469) | (474/474)/(494/494) | (454/454)/(587/587) | (323/479)/(516/516) |
| AF511039.1\| FMDV - type O strain Akesu/58 | (458/458)/(331/469) | (474/474)/(482/482) | (381/454)/(321/587) | (444/496)/(465/465) |
| HQ832592.1\| FMDV - type A isolate IND 17/2009 | (458/458)/(469/469) | (474/474)/(482/482) | (454/454)/(587/587) | (487/487)/(444/444) |
| HQ832591.1\| FMDV - type A isolate IND 437/2008 | (392/458)/(469/469) | (460/460)/(482/482) | (454/454)/(587/587) | (487/487)/(444/444) |
| HQ832590.1\| FMDV - type A isolate IND 245/2007 | (458/458)/(469/469) | (474/474)/(482/482) | (454/454)/(587/587) | (487/487)/(444/444) |
| HQ832589.1\| FMDV - type A isolate IND 109/2006 | (458/458)/(415/469) | (474/474)/(482/482) | (454/454)/(587/587) | (487/487)/(466/466) |
| HQ832588.1\| FMDV - type A isolate IND 88/2006 | (458/458)/(415/469) | (474/474)/(482/482) | (454/454)/(587/587) | (419/487)/(280/466) |
| HQ832587.1\| FMDV - type A isolate IND 50/2006 | (458/458)/(469/469) | (474/474)/(482/482) | (454/454)/(587/587) | (487/487)/(444/444) |
| HQ832586.1\| FMDV - type A isolate IND 43/2006 | (458/458)/(469/469) | (474/474)/(482/482) | (454/454)/(587/587) | (496/496)/(516/516) |
| HQ832585.1\| FMDV - type A isolate IND 26/2006 | (458/458)/(469/469) | (474/474)/(482/482) | (454/454)/(587/587) | (487/487)/(516/516) |
| HQ832584.1\| FMDV - type A isolate IND 22/2006 | (458/458)/(397/469) | (474/474)/(482/482) | (313/454)/(587/587) | (487/487)/(516/516) |
| HQ832583.1\| FMDV - type A isolate IND 447/2005 | (458/458)/(397/469) | (474/474)/(482/482) | (313/454)/(587/587) | (467/487)/(507/507) |
| HQ832582.1\| FMDV - type A isolate IND 249/2004 | (458/458)/(469/469) | (474/474)/(482/482) | (454/454)/(587/587) | (394/487)/(516/516) |
| HQ832581.1\| FMDV - type A isolate IND 64/2004 | (458/458)/(469/469) | (474/474)/(482/482) | (454/454)/(587/587) | (409/487)/(516/516) |
| HQ832580.1\| FMDV - type A isolate IND 818/2003 | (458/458)/(469/469) | (474/474)/(482/482) | (454/454)/(587/587) | (487/487)/(516/516) |
| HQ832579.1\| FMDV - type A isolate IND 281/2003 | (458/458)/(469/469) | (474/474)/(482/482) | (454/454)/(587/587) | (487/487)/(516/516) |
| HQ832578.1\| FMDV - type A isolate IND 161/2003 | (458/458)/(469/469) | (474/474)/(482/482) | (454/454)/(587/587) | (487/487)/(516/516) |
| HQ832577.1\| FMDV - type A isolate IND 110/1999 | (458/458)/(469/469) | (474/474)/(482/482) | (454/454)/(587/587) | (492/492)/(516/516) |
| HQ832576.1\| FMDV - type A isolate IND 21/1990 | (458/458)/(469/469) | (474/474)/(482/482) | (176/454)/(587/587) | (487/487)/(445/516) |
| KC412634.1\| FMDV - type Asia 1 isolate Asia1/HN/2006 | (178/458)/(469/469) | (326/474)/(482/482) | (454/454)/(587/587) | (487/487)/(516/516) |
| HM854024.1\| FMDV - type A strain IND17/82 | (458/458)/(469/469) | (474/474)/(482/482) | (454/454)/(587/587) | (487/487)/(516/516) |
| HM854023.1\| FMDV - type A strain IND258/99 | (458/458)/(469/469) | (474/474)/(482/482) | (454/454)/(587/587) | (487/487)/(516/516) |
| HM854022.1\| FMDV - type A strain IND17/77 | (458/458)/(469/469) | (474/474)/(482/482) | (377/454)/(587/587) | (350/487)/(466/466) |
| HM854021.1\| FMDV - type A strain IND81/00 | (458/458)/(469/469) | (474/474)/(482/482) | (454/454)/(587/587) | (487/487)/(516/516) |
| DQ533483.2\| FMDV - type Asia 1 strain ZB/CHA/58 | (458/458)/(469/469) | (474/474)/(482/482) | (454/454)/(587/587) | (487/487)/(444/444) |
| JX040501.1\| FMDV - type O isolate ISR/2/2011 | (392/458)/(469/469) | (460/460)/(455/482) | (454/454)/(292/587) | (350/487)/(466/466) |
| JX040500.1\| FMDV - type O isolate TUR/27/2011 | (316/458)/(469/469) | (361/474)/(482/482) | (454/454)/(587/587) | (391/487)/(466/466) |
| JX040499.1\| FMDV - type O isolate TUR/8/2011 | (458/458)/(469/469) | (474/474)/(482/482) | (454/454)/(587/587) | (391/487)/(444/444) |
| JX040498.1\| FMDV - type O isolate TUR/1086/2010 | (392/458)/(469/469) | (460/460)/(482/482) | (454/454)/(587/587) | (391/487)/(466/466) |
| JX040497.1\| FMDV - type O isolate TUR/1003/2010 | (458/458)/(469/469) | (474/474)/(482/482) | (407/454)/(587/587) | (391/487)/(466/466) |
| JX040496.1\| FMDV - type O isolate TUR/926/2010 | (458/458)/(469/469) | (474/474)/(482/482) | (454/454)/(587/587) | (391/487)/(466/466) |
| JX040495.1\| FMDV - type O isolate TUR/883/2010 | (458/458)/(469/469) | (474/474)/(482/482) | (454/454)/(587/587) | (391/487)/(466/466) |
| JX040494.1\| FMDV - type O isolate TUR/868/2010 | (458/458)/(469/469) | (474/474)/(482/482) | (454/454)/(587/587) | (391/487)/(466/466) |
| JX040493.1\| FMDV - type O isolate TUR/840/2010 | (458/458)/(469/469) | (474/474)/(482/482) | (454/454)/(587/587) | (391/487)/(466/466) |
| JX040492.1\| FMDV - type O isolate TUR/36/2010 | (392/458)/(469/469) | (460/460)/(482/482) | (454/454)/(587/587) | (391/487)/(466/466) |
| JX040491.1\| FMDV - type O isolate TUR/18/2010 | (458/458)/(469/469) | (474/474)/(482/482) | (454/454)/(587/587) | (391/487)/(466/466) |
| JX040490.1\| FMDV - type O isolate BUL/32/2011 | (458/458)/(469/469) | (474/474)/(482/482) | (454/454)/(587/587) | (362/487)/(390/444) |
| JX040489.1\| FMDV - type O isolate BUL/30/2011 | (458/458)/(469/469) | (474/474)/(482/482) | (454/454)/(587/587) | (362/487)/(390/444) |
| JX040488.1\| FMDV - type O isolate BUL/26/2011 | (458/458)/(469/469) | (474/474)/(482/482) | (454/454)/(587/587) | (362/487)/(390/444) |
| JX040487.1\| FMDV - type O isolate BUL/20/2011 | (458/458)/(469/469) | (474/474)/(482/482) | (454/454)/(587/587) | (362/487)/(390/444) |
| JX040486.1\| FMDV - type O isolate BUL/11/2011 | (458/458)/(469/469) | (474/474)/(482/482) | (175/454)/(587/587) | (362/487)/(390/444) |
| JX040485.1\| FMDV - type O isolate BUL/1/2010 | (458/458)/(469/469) | (474/474)/(482/482) | (454/454)/(587/587) | (375/487)/(390/444) |
| JX066665.1\| FMDV - type O isolate 12LPN3 | (458/458)/(469/469) | (474/474)/(482/482) | (454/454)/(587/587) | (375/487)/(390/444) |
| JX066664.1\| FMDV - type O isolate 12LPN1 | (458/458)/(469/469) | (474/474)/(482/482) | (454/454)/(587/587) | (375/487)/(390/444) |
| HM008917.1\| FMDV - type O strain O/YS/CHA/05 | (458/458)/(469/469) | (474/474)/(482/482) | (454/454)/(587/587) | (487/487)/(516/516) |
| EF614457.1\| FMDV - type O strain O/SKR/14/02 | (458/458)/(469/469) | (474/474)/(482/482) | (454/454)/(587/587) | (487/487)/(516/516) |
| EF614458.1\| FMDV - type Asia 1 strain Asia1/MOG/05 | (178/458)/(469/469) | (326/474)/(482/482) | (454/454)/(587/587) | (487/487)/(516/516) |
| 21426907\|ref\|NC_004004.1\| FMDV - type O | (458/458)/(469/469) | (474/474)/(482/482) | (454/454)/(583/583) | (504/504)/(474/474) |
| JX570655.1\| FMDV - type O isolate BFS 1860 B5.9D.V | (458/458)/(469/469) | (474/474)/(482/482) | (454/454)/(587/587) | (487/487)/(466/466) |
| JX570654.1\| FMDV - type O isolate BFS 1860 B4.9D.V | (458/458)/(469/469) | (474/474)/(482/482) | (454/454)/(587/587) | (487/487)/(466/466) |
| JX570653.1\| FMDV - type O isolate BFS 1860 B3.3D.V | (458/458)/(469/469) | (474/474)/(482/482) | (454/454)/(587/587) | (487/487)/(466/466) |
| JX570652.1\| FMDV - type O isolate BFS 1860 B2.32D.P | (458/458)/(469/469) | (474/474)/(482/482) | (454/454)/(587/587) | (487/487)/(466/466) |
| JX570651.1\| FMDV - type O isolate BFS 1860 B2.6D.P | (458/458)/(469/469) | (474/474)/(482/482) | (454/454)/(587/587) | (487/487)/(466/466) |
| JX570650.1\| FMDV - type O isolate BFS 1860 B2.6D.V | (458/458)/(469/469) | (474/474)/(482/482) | (454/454)/(587/587) | (487/487)/(466/466) |
| JX570649.1\| FMDV - type O isolate BFS 1860 B2.4D.P | (458/458)/(469/469) | (474/474)/(482/482) | (454/454)/(587/587) | (487/487)/(466/466) |
| JX570648.1\| FMDV - type O isolate BFS 1860 B2.2D.P | (458/458)/(469/469) | (474/474)/(482/482) | (454/454)/(587/587) | (487/487)/(466/466) |
| JX570647.1\| FMDV - type O isolate BFS 1860 B1.2D.V | (458/458)/(469/469) | (474/474)/(482/482) | (454/454)/(587/587) | (487/487)/(466/466) |
| JX570646.1\| FMDV - type O isolate BFS 1860 A5.9D.V | (458/458)/(469/469) | (474/474)/(482/482) | (454/454)/(587/587) | (487/487)/(466/466) |
| JX570645.1\| FMDV - type O isolate BFS 1860 A4.12D.V | (458/458)/(469/469) | (474/474)/(482/482) | (454/454)/(587/587) | (487/487)/(466/466) |
| JX570644.1\| FMDV - type O isolate BFS 1860 A3.5D.V | (458/458)/(469/469) | (474/474)/(482/482) | (454/454)/(587/587) | (487/487)/(466/466) |
| JX570643.1\| FMDV - type O isolate BFS 1860 A2.32D.P | (458/458)/(469/469) | (474/474)/(482/482) | (454/454)/(587/587) | (487/487)/(466/466) |
| JX570642.1\| FMDV - type O isolate BFS 1860 A2.6D.P | (458/458)/(469/469) | (474/474)/(482/482) | (454/454)/(587/587) | (487/487)/(466/466) |
| JX570641.1\| FMDV - type O isolate BFS 1860 A2.6D.V | (458/458)/(469/469) | (474/474)/(482/482) | (454/454)/(587/587) | (487/487)/(466/466) |
| JX570640.1\| FMDV - type O isolate BFS 1860 A2.4D.P | (458/458)/(469/469) | (474/474)/(482/482) | (454/454)/(587/587) | (487/487)/(466/466) |
| JX570639.1\| FMDV - type O isolate BFS 1860 A2.2D.P | (458/458)/(469/469) | (474/474)/(482/482) | (454/454)/(587/587) | (487/487)/(466/466) |
| JX570638.1\| FMDV - type O isolate BFS 1860 A1.2D.V | (458/458)/(469/469) | (474/474)/(482/482) | (454/454)/(587/587) | (487/487)/(466/466) |
| JN998086.1\| FMDV - type O strain O/GZ/CHA/2010 | (458/458)/(415/469) | (474/474)/(482/482) | (454/454)/(587/587) | (391/487)/(468/516) |
| JN998085.1\| FMDV - type O strain O/BY/CHA/2010 | (458/458)/(415/469) | (474/474)/(482/482) | (454/454)/(587/587) | (391/487)/(468/516) |
| HQ631363.1\| FMDV - type Asia 1 isolate Asia1/1/YZ/CHA/06 | (178/458)/(469/469) | (326/474)/(482/482) | (454/454)/(587/587) | (487/487)/(516/516) |
| JF739177.1\| FMDV - type Asia 1 isolate As1/Shamir/89 | (458/458)/(469/469) | (474/474)/(482/482) | (454/454)/(587/587) | (487/487)/(516/516) |
| HQ632774.1\| FMDV - type Asia 1 isolate MAY/9/99 | (432/458)/(469/469) | (449/474)/(482/482) | (353/454)/(587/587) | (419/487)/(516/516) |
| HQ632773.1\| FMDV - type A isolate MAY/3/2007 | (458/458)/(469/469) | (474/474)/(482/482) | (454/454)/(583/583) | (496/496)/(516/516) |
| HQ632772.1\| FMDV - type O isolate MAY/7/2007 | (458/458)/(469/469) | (474/474)/(482/482) | (454/454)/(587/587) | (487/487)/(516/516) |
| HQ632771.1\| FMDV - type O isolate MAY/8/2005 | (458/458)/(469/469) | (474/474)/(482/482) | (419/454)/(583/583) | (383/492)/(516/516) |
| HQ632770.1\| FMDV - type O isolate MAY/1/2004 | (458/458)/(469/469) | (474/474)/(482/482) | (454/454)/(587/587) | (487/487)/(516/516) |
| HQ632769.1\| FMDV - type O isolate MAY/7/2001 | (458/458)/(469/469) | (474/474)/(482/482) | (454/454)/(587/587) | (447/487)/(516/516) |
| HQ632768.1\| FMDV - type O isolate MAY/3/2000 | (458/458)/(469/469) | (474/474)/(482/482) | (454/454)/(587/587) | (487/487)/(516/516) |
| FJ906802.1\| FMDV - type Asia 1 strain Asia1/WHN/CHA/06 | (178/458)/(469/469) | (326/474)/(482/482) | (454/454)/(587/587) | (499/499)/(516/516) |
| HQ412603.1\| FMDV - type O strain O/YM/YN/2000 | (458/458)/(469/469) | (474/474)/(482/482) | (381/454)/(583/583) | (477/504)/(474/474) |
| HM854025.1\| FMDV - type A strain IND40/00 | (458/458)/(469/469) | (474/474)/(482/482) | (454/454)/(587/587) | (487/487)/(516/516) |
| JX014256.1\| FMDV - type SAT 2 isolate PAT/1/2012 | (416/458)/(469/469) | (434/474)/(482/482) | (316/454)/(587/587) | (341/492)/(381/444) |
| JX014255.1\| FMDV - type SAT 2 isolate EGY/9/2012 | (361/458)/(469/469) | (420/460)/(482/482) | (316/454)/(587/587) | (341/492)/(381/444) |
| GU384683.1\| FMDV - type O isolate PAK/45/2008 | (458/458)/(469/469) | (474/474)/(482/482) | (454/454)/(587/587) | (487/487)/(516/516) |
| GU384682.1\| FMDV - type O isolate PAK/44/2008 | (458/458)/(469/469) | (474/474)/(482/482) | (454/454)/(587/587) | (487/487)/(516/516) |
| FJ175666.1\| FMDV - type O isolate Israel 07-6387 | (458/458)/(255/469) | (474/474)/(482/482) | (454/454)/(587/587) | (430/496)/(516/516) |
| FJ175665.1\| FMDV - type O isolate Israel 07-6391 | (458/458)/(255/469) | (474/474)/(482/482) | (454/454)/(587/587) | (430/496)/(516/516) |
| FJ175664.1\| FMDV - type O isolate Israel 07-6389 | (458/458)/(469/469) | (474/474)/(482/482) | (454/454)/(587/587) | (430/496)/(516/516) |
| FJ175663.1\| FMDV - type O isolate Israel 07-6382 | (458/458)/(469/469) | (474/474)/(482/482) | (454/454)/(587/587) | (430/496)/(516/516) |
| FJ175662.1\| FMDV - type O isolate Israel 07-6380 | (458/458)/(255/469) | (474/474)/(482/482) | (454/454)/(587/587) | (430/496)/(516/516) |
| FJ175661.1\| FMDV - type O isolate Israel 07-6378 | (458/458)/(255/469) | (474/474)/(482/482) | (454/454)/(587/587) | (430/496)/(516/516) |
| AY686687.1\| FMDV O/ES/2001 isolate O/ES/2001 | (458/458)/(469/469) | (474/474)/(482/482) | (353/454)/(587/587) | (504/504)/(474/474) |
| DQ989322.1\| FMDV - type Asia 1 isolate IND 139-02 | (392/458)/(469/469) | (460/460)/(482/482) | (454/454)/(371/587) | (487/487)/(466/466) |
| EF149010.1\| FMDV - type Asia 1 strain Asia 1/HNK/CHA/05 | (335/458)/(469/469) | (371/474)/(482/482) | (454/454)/(587/587) | (487/487)/(466/466) |
| KF112889.1\| FMDV - type O isolate HKN15/2010 | (458/458)/(415/469) | (474/474)/(482/482) | (454/454)/(587/587) | (391/487)/(468/516) |
| KF112888.1\| FMDV - type O isolate DRK/31/2011 | (458/458)/(415/469) | (474/474)/(482/482) | (454/454)/(587/587) | (350/487)/(468/516) |
| KF112887.1\| FMDV - type O isolate SKR/5/2010 | (458/458)/(415/469) | (474/474)/(482/482) | (454/454)/(587/587) | (391/487)/(468/516) |
| KF112886.1\| FMDV - type O isolate SKR/4/2010 | (458/458)/(415/469) | (474/474)/(482/482) | (454/454)/(587/587) | (391/487)/(468/516) |
| KF112885.1\| FMDV - type O isolate JPN/1/2010 | (458/458)/(415/469) | (474/474)/(482/482) | (454/454)/(587/587) | (391/487)/(468/516) |
| KF112884.1\| FMDV - type O isolate RUS/Aug 2010 | (458/458)/(469/469) | (474/474)/(482/482) | (454/454)/(587/587) | (409/487)/(516/516) |
| KF112883.1\| FMDV - type O isolate RUS/Jul 2010 | (458/458)/(415/469) | (474/474)/(482/482) | (454/454)/(587/587) | (391/487)/(468/516) |
| KF112882.1\| FMDV - type O isolate MOG/C-10/2010 | (458/458)/(469/469) | (474/474)/(417/482) | (454/454)/(587/587) | (409/487)/(516/516) |
| KF112881.1\| FMDV - type O isolate MOG/7/2010 | (458/458)/(469/469) | (474/474)/(482/482) | (454/454)/(587/587) | (409/487)/(516/516) |
| KF112880.1\| FMDV - type O isolate MYA/5/2009 | (458/458)/(469/469) | (474/474)/(482/482) | (454/454)/(587/587) | (350/487)/(468/516) |
| KF112879.1\| FMDV - type O isolate TAI/22/2009 | (458/458)/(469/469) | (474/474)/(482/482) | (454/454)/(587/587) | (396/496)/(516/516) |
| JF749862.1\| FMDV - type SAT 2 isolate UGA_002/2002 | (458/458)/(469/469) | (474/474)/(482/482) | (454/454)/(587/587) | (363/492)/(373/440) |
| JF749860.1\| FMDV - type SAT 1 isolate KEN_004/2002 | (458/458)/(255/469) | (474/474)/(482/482) | (454/454)/(587/587) | (348/501)/(466/466) |
| JF749851.1\| FMDV - type O isolate IRN_073/2001 | (458/458)/(469/469) | (474/474)/(482/482) | (454/454)/(587/587) | (487/487)/(516/516) |
| JF749843.1\| FMDV - type A isolate Egypt/2006 | (458/458)/(469/469) | (474/474)/(494/494) | (454/454)/(587/587) | (399/501)/(394/461) |
| JX869188.1\| FMDV - type O isolate BFS 89/68 | (458/458)/(469/469) | (474/474)/(482/482) | (454/454)/(587/587) | (487/487)/(466/466) |
| JX869187.1\| FMDV - type O isolate BFS 86/68 | (458/458)/(469/469) | (474/474)/(482/482) | (454/454)/(587/587) | (487/487)/(466/466) |
| JX869186.1\| FMDV - type O isolate BFS 69/68 | (458/458)/(469/469) | (474/474)/(482/482) | (454/454)/(587/587) | (487/487)/(466/466) |
| JX869185.1\| FMDV - type O isolate BFS 63/68 | (458/458)/(469/469) | (474/474)/(482/482) | (396/454)/(587/587) | (487/487)/(466/466) |
| JX869184.1\| FMDV - type O isolate BFS 45/68 | (458/458)/(469/469) | (474/474)/(482/482) | (454/454)/(587/587) | (487/487)/(466/466) |
| JX869183.1\| FMDV - type O isolate BFS 41/68 | (458/458)/(469/469) | (474/474)/(482/482) | (454/454)/(587/587) | (487/487)/(466/466) |
| JX869182.1\| FMDV - type O isolate BFS 11/68 | (458/458)/(469/469) | (474/474)/(482/482) | (454/454)/(587/587) | (487/487)/(466/466) |
| JX869181.1\| FMDV - type O isolate BFS 1950 | (458/458)/(469/469) | (474/474)/(482/482) | (454/454)/(587/587) | (487/487)/(466/466) |
| JX869180.1\| FMDV - type O isolate BFS 1889 | (458/458)/(469/469) | (474/474)/(482/482) | (454/454)/(587/587) | (487/487)/(466/466) |
| JX869179.1\| FMDV - type O isolate BFS 1848 | (458/458)/(469/469) | (474/474)/(482/482) | (454/454)/(587/587) | (487/487)/(466/466) |
| JX869178.1\| FMDV - type O isolate BFS 1836 | (458/458)/(469/469) | (474/474)/(482/482) | (454/454)/(587/587) | (487/487)/(466/466) |
| JX869177.1\| FMDV - type O isolate BFS 1810A | (458/458)/(469/469) | (474/474)/(482/482) | (454/454)/(587/587) | (487/487)/(466/466) |
| JQ900581.1\| FMDV - type O strain O/GSLX/2010 | (458/458)/(415/469) | (474/474)/(482/482) | (454/454)/(587/587) | (391/487)/(468/516) |
| GU931682.1\| FMDV - type Asia 1 isolate Asia1/YS/CHA/05 | (178/458)/(469/469) | (326/474)/(482/482) | (454/454)/(587/587) | (487/487)/(516/516) |
| HQ009509.1\| FMDV - type O strain China/5/99(Fujian) | (432/458)/(415/469) | (449/474)/(482/482) | (454/454)/(587/587) | (487/487)/(516/516) |
| EU214601.1\| FMDV - type O strain UKG/8098/2001 | (458/458)/(469/469) | (474/474)/(482/482) | (454/454)/(587/587) | (487/487)/(516/516) |
| EF552697.1\| FMDV O/UKG/5681/2001 | (458/458)/(469/469) | (474/474)/(482/482) | (454/454)/(587/587) | (419/487)/(516/516) |
| EF552696.1\| FMDV O/UKG/5470/2001 | (458/458)/(469/469) | (474/474)/(482/482) | (454/454)/(587/587) | (487/487)/(516/516) |
| EF552695.1\| FMDV O/UKG/9443/2001 | (458/458)/(469/469) | (474/474)/(482/482) | (454/454)/(587/587) | (487/487)/(516/516) |
| EF552694.1\| FMDV O/UKG/4998/2001 | (458/458)/(469/469) | (474/474)/(482/482) | (454/454)/(587/587) | (487/487)/(516/516) |
| EF552693.1\| FMDV O/UKG/4014/2001 | (458/458)/(469/469) | (474/474)/(482/482) | (454/454)/(587/587) | (487/487)/(516/516) |
| EF552692.1\| FMDV O/UKG/7299/2001 | (458/458)/(469/469) | (474/474)/(482/482) | (454/454)/(587/587) | (487/487)/(516/516) |
| EF552691.1\| FMDV O/UKG/9161/2001 | (458/458)/(469/469) | (474/474)/(482/482) | (454/454)/(587/587) | (487/487)/(516/516) |
| EF552690.1\| FMDV O/UKG/7039/2001 | (458/458)/(469/469) | (474/474)/(482/482) | (454/454)/(587/587) | (487/487)/(516/516) |
| EF552689.1\| FMDV O/UKG/4141/2001 | (458/458)/(469/469) | (474/474)/(482/482) | (454/454)/(587/587) | (487/487)/(516/516) |
| EF552688.1\| FMDV O/UKG/3952/2001 | (458/458)/(469/469) | (474/474)/(482/482) | (454/454)/(587/587) | (487/487)/(516/516) |
| DQ989323.1\| FMDV - type Asia 1 isolate IND 97-03 | (458/458)/(469/469) | (474/474)/(482/482) | (153/454)/(587/587) | (487/487)/(466/466) |
| DQ989321.1\| FMDV - type Asia 1 isolate IND 438-01 | (458/458)/(469/469) | (474/474)/(482/482) | (454/454)/(587/587) | (487/487)/(466/466) |
| DQ989320.1\| FMDV - type Asia 1 isolate IND 182-02 | (458/458)/(469/469) | (474/474)/(482/482) | (454/454)/(587/587) | (487/487)/(466/466) |
| DQ989319.1\| FMDV - type Asia 1 isolate IND 423-01 | (458/458)/(469/469) | (474/474)/(482/482) | (454/454)/(587/587) | (487/487)/(466/466) |
| DQ989318.1\| FMDV - type Asia 1 isolate IND 61-02 | (458/458)/(469/469) | (474/474)/(482/482) | (454/454)/(583/583) | (487/487)/(466/466) |
| DQ989317.1\| FMDV - type Asia 1 isolate IND 148-01 | (458/458)/(469/469) | (474/474)/(482/482) | (454/454)/(587/587) | (487/487)/(466/466) |
| DQ989316.1\| FMDV - type Asia 1 isolate IND 21-89 | (458/458)/(469/469) | (474/474)/(482/482) | (454/454)/(587/587) | (496/496)/(280/431) |
| DQ989315.1\| FMDV - type Asia 1 isolate IND 47-93 | (458/458)/(469/469) | (474/474)/(442/482) | (454/454)/(479/587) | (487/487)/(516/516) |
| DQ989314.1\| FMDV - type Asia 1 isolate IND 354-01 | (458/458)/(469/469) | (474/474)/(482/482) | (454/454)/(587/587) | (487/487)/(466/466) |
| DQ989313.1\| FMDV - type Asia 1 isolate IND 52-87 | (458/458)/(469/469) | (474/474)/(482/482) | (353/454)/(587/587) | (351/487)/(516/516) |
| DQ989312.1\| FMDV - type Asia 1 isolate IND 13-91 | (458/458)/(469/469) | (474/474)/(482/482) | (454/454)/(587/587) | (487/487)/(243/431) |
| DQ989311.1\| FMDV - type Asia 1 isolate IND 37-02 | (458/458)/(469/469) | (474/474)/(482/482) | (454/454)/(587/587) | (447/487)/(516/516) |
| DQ989310.1\| FMDV - type Asia 1 isolate IND 101-99 | (458/458)/(469/469) | (474/474)/(482/482) | (454/454)/(301/587) | (487/487)/(516/516) |
| DQ989309.1\| FMDV - type Asia 1 isolate IND 82-96 | (458/458)/(469/469) | (474/474)/(482/482) | (454/454)/(587/587) | (496/496)/(516/516) |
| DQ989308.1\| FMDV - type Asia 1 isolate IND 397-97 | (458/458)/(469/469) | (474/474)/(482/482) | (454/454)/(587/587) | (496/496)/(466/466) |
| DQ989307.1\| FMDV - type Asia 1 isolate IND 247-92 | (458/458)/(469/469) | (474/474)/(482/482) | (454/454)/(371/587) | (487/487)/(253/466) |
| DQ989306.1\| FMDV - type Asia 1 isolate IND 81-86 | (458/458)/(469/469) | (474/474)/(482/482) | (454/454)/(587/587) | (487/487)/(516/516) |
| DQ989305.1\| FMDV - type Asia 1 isolate IND 116-90 | (392/458)/(469/469) | (460/460)/(482/482) | (454/454)/(587/587) | (487/487)/(253/466) |
| DQ989304.1\| FMDV - type Asia 1 isolate IND 334-00 | (458/458)/(469/469) | (474/474)/(482/482) | (454/454)/(587/587) | (487/487)/(516/516) |
| DQ989303.1\| FMDV - type Asia 1 isolate IND 151-94 | (458/458)/(469/469) | (474/474)/(482/482) | (454/454)/(587/587) | (487/487)/(516/516) |
| FJ824812.1\| FMDV - type C strain C-S8p200 | (458/458)/(469/469) | (474/474)/(494/494) | (454/454)/(587/587) | (323/479)/(516/516) |
| HM229661.1\| FMDV - type O isolate HKN/20/2010 | (458/458)/(415/469) | (474/474)/(482/482) | (454/454)/(587/587) | (391/487)/(468/516) |
| EF149009.1\| FMDV - type Asia 1 strain Asia 1/Jiangsu/China/2005 | (141/458)/(469/469) | (279/474)/(482/482) | (454/454)/(587/587) | (499/499)/(516/516) |
| KC503937.1\| FMDV - type O strain Andong | (458/458)/(415/469) | (474/474)/(482/482) | (454/454)/(587/587) | (391/487)/(468/516) |
| FJ542372.1\| FMDV - type O isolate UKG/2640/2001 | (458/458)/(469/469) | (474/474)/(482/482) | (454/454)/(587/587) | (487/487)/(516/516) |
| FJ542371.1\| FMDV - type O isolate UKG/2526/2001 | (458/458)/(469/469) | (474/474)/(482/482) | (454/454)/(587/587) | (487/487)/(516/516) |
| FJ542370.1\| FMDV - type O isolate UKG/2085/2001 | (458/458)/(469/469) | (474/474)/(482/482) | (454/454)/(587/587) | (487/487)/(516/516) |
| FJ542369.1\| FMDV - type O isolate UKG/2000/2001 | (458/458)/(469/469) | (474/474)/(482/482) | (454/454)/(587/587) | (487/487)/(516/516) |
| FJ542368.1\| FMDV - type O isolate UKG/1734/2001 | (458/458)/(469/469) | (474/474)/(482/482) | (454/454)/(587/587) | (487/487)/(516/516) |
| FJ542367.1\| FMDV - type O isolate UKG/1558/2001 | (458/458)/(469/469) | (474/474)/(482/482) | (454/454)/(587/587) | (487/487)/(516/516) |
| FJ542366.1\| FMDV - type O isolate UKG/1450/2001 | (458/458)/(469/469) | (474/474)/(482/482) | (454/454)/(587/587) | (487/487)/(516/516) |
| FJ542365.1\| FMDV - type O isolate UKG/417/2001 | (458/458)/(469/469) | (474/474)/(482/482) | (454/454)/(587/587) | (487/487)/(516/516) |
| KC462884.1\| FMDV - type Asia 1 isolate Asial/HN/2006 | (178/458)/(469/469) | (326/474)/(482/482) | (454/454)/(587/587) | (487/487)/(516/516) |

**TableS3.** Repeatability intra-assay for the mMulti-rRT-PCR assessed on the different TgRs.

|  | | **mean Ct±SD** | | | **CV** |
| --- | --- | --- | --- | --- | --- |
|  |  | **[RNA]=10^2^** | **[RNA]=10^4^** | **[RNA]=10^6^** |  |
| **mMulti-rRT-PCR assay** | **TgR1** | 16.17±0.04 | 24.24±0.06 | 32.58±0.17 | 0.20 %-0.50 % |
|  | **TgR2** | 15.4±0.02 | 22.29±0.05 | 29.17±0.06 | 0.12 %-0.23 % |
|  | **TgR3** | 13.29±0.03 | 20.68±0.06 | 27.91±0.06 | 0.20 %-0.27 % |
|  | **TgR4** | 11.38±0.04 | 22.19±0.04 | 29.65±0.07 | 0.21 %-0.34 % |

**[RNA]:** Concentration of RNA expressed by copy number/µL.


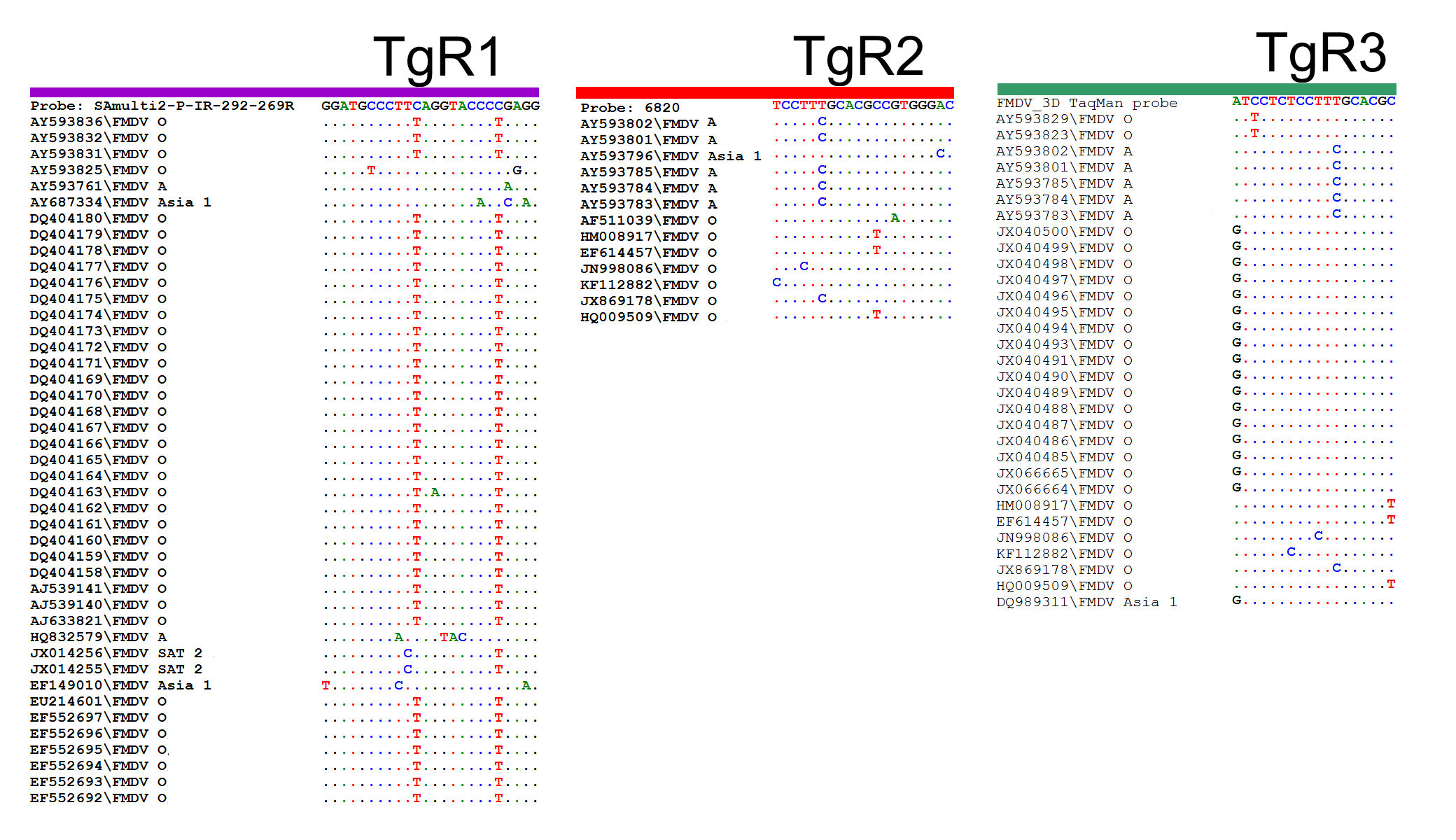


**Fig. S1. Alignment of sequences and probe binding regions** A) Probe matching TgR1 B) Probe matching TgR2, C) Probe matching TgR3. Those sequences with one mismatch between probe and target sequence (for TgR2 and TgR3) or two mismatches or more for TgR1 are shown.


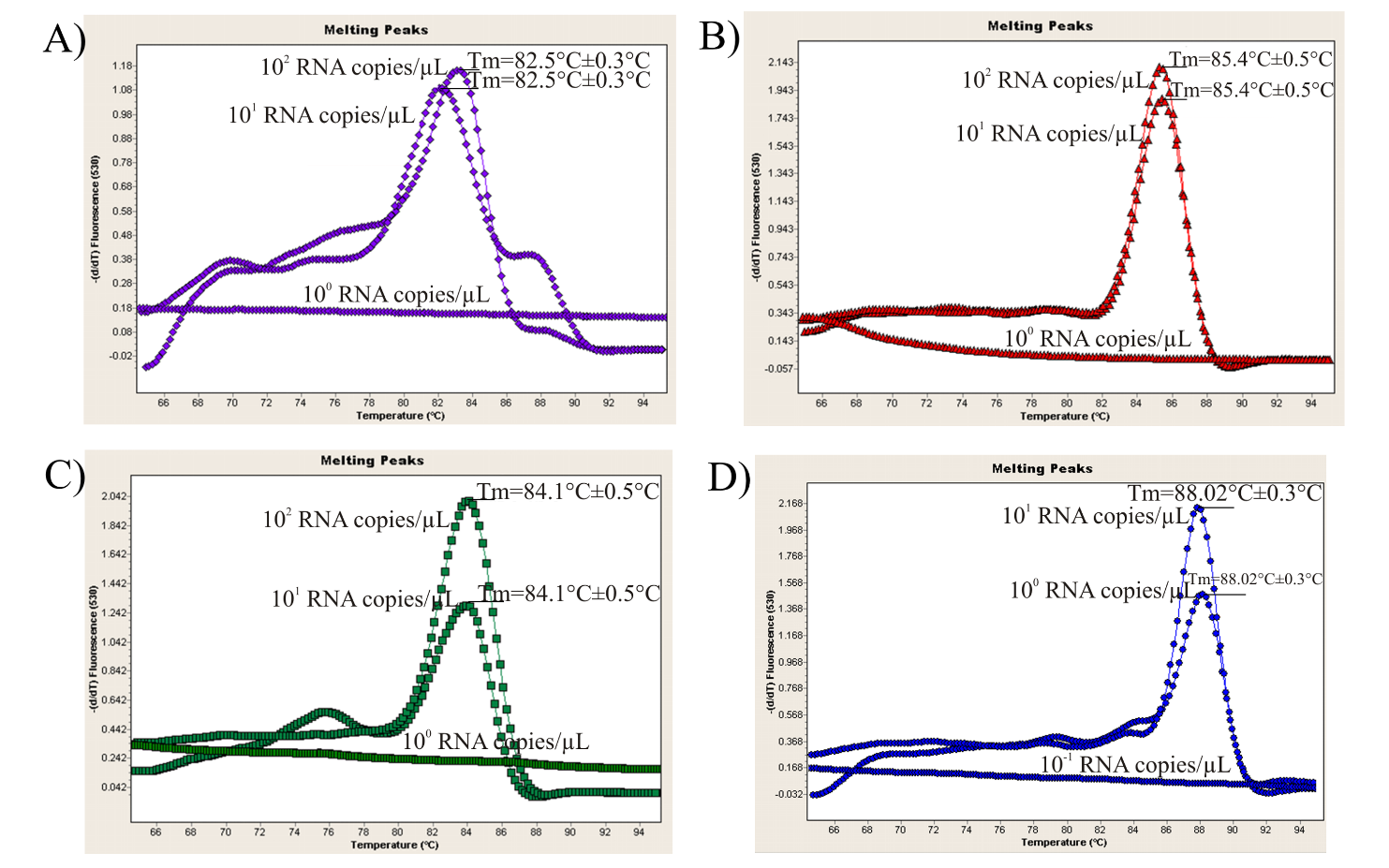


**Fig. S2. Detection limit of the mMulti-rRT-PCR assay.** The detection limit was determined based on serial ten-fold dilutions of *in-vitro* transcribed viral RNA for each TgR. A) TgR1, B) TgR2, C) TgR3 and D) TgR4. The limit of detection was determined as the last dilution reliably detected with a specific melting curve. The specific Tm and melting peak for each TgR is also shown.


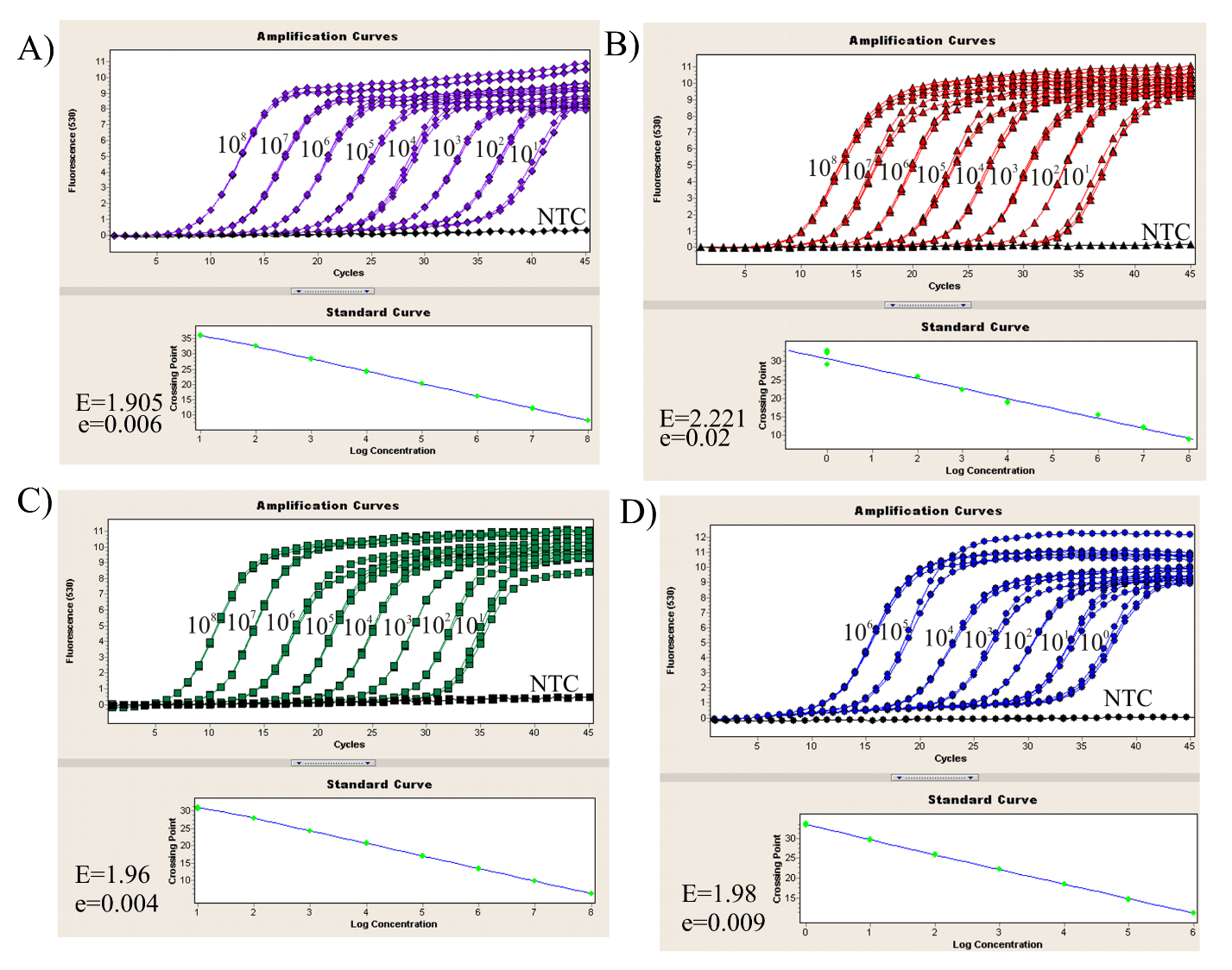


**Fig. S3. Standard curve of the mMulti-rRT-PCR assay. Serial ten-fold dilutions of *in vitro* transcribed viral RNA for each TgR is shown**. A) TgR1, B) TgR2, C) TgR3 and D) TgR4. Amplification curves evaluation of serial dilutions (10-fold) of *in vitro* transcribed RNA are shown. The standard curve shows the linear range of the assays for each target on the dilutions assessed. The efficiency value (E) and standard error associated to each reaction are also shown.
